# Mechanical deformation explains distinct neuroimaging patterns and etiologies in brain trauma

**DOI:** 10.1016/j.neuroimage.2026.122088

**Published:** 2026-07-01

**Authors:** Yuan Huang, Tianjia Zhu, Ishfaque Ahmed, Morgan LaBalle, Qun Zhao, Moira F. Taber, Sydney E. Sneed, Erin E. Kaiser, Franklin D. West, Kevin D. Browne, D.Kacy Cullen, David F. Meaney, Taotao Wu

**Affiliations:** a School of Chemical, Materials and Biomedical Engineering, University of Georgia, Athens, GA, United States; b International Center for Automotive Medicine (ICAM), University of Michigan, Ann Arbor, MI, United States; c Departments of Bioengineering, University of Pennsylvania, Philadelphia, PA, United States; d Department of Radiology, Children’s Hospital of Philadelphia, Philadelphia, PA, United States; e Department of Physics and Astronomy, University of Georgia, Athens, GA, United States; f Regenerative Bioscience Center, University of Georgia, Athens, GA, United States; g Department of Animal and Dairy Science, University of Georgia, Athens, GA, United States; h Center for Brain Injury & Repair, Department of Neurosurgery, University of Pennsylvania, Philadelphia, PA, United States; i Center for Neurotrauma, Neurodegeneration, & Restoration, Corporal Michael Crescenz VA Medical Center, Philadelphia, PA, United States

**Keywords:** Finite element model, Preclinical pig model, Controlled cortical impact, Non-impact rotational injury, Traumatic brain injury, Diffusion magnetic resonance imaging

## Abstract

Understanding how mechanical forces translate into progressive neural pathology remains a central challenge in mitigating, diagnosing, and treating traumatic brain injury (TBI), a major public health concern that affects millions annually and lacks reliable prognostic biomarkers. To address this, we combined virtual brain twins constructed using finite element methods with diffusion imaging to investigate spatiotemporal tissue alterations in two porcine TBI models: non-impact rotational TBI and controlled cortical impact (CCI). In the rotational model, subacute abnormalities were observed in the brainstem and subcortical regions, with quantitative anisotropy showing a moderate correlation with peak strain. In contrast, CCI produced delayed, but widespread, diffusion abnormalities, particularly in mean diffusivity, that were associated with cortical deformation over time. Axonal injury metrics derived from tractography-based structural connectivity showed strain-related trends in the rotational model but not in CCI, suggesting that diffuse axonal injury from inertial loading may be more directly influenced by mechanical factors, whereas axonal damage in focal impact may be more influenced by secondary, non-mechanical processes, such as neuroinflammation. These findings support a mechanistic association between simulated brain deformation and evolving imaging biomarkers, indicating that different TBI mechanisms (diffuse versus focal) are associated with distinct spatiotemporal diffusion signatures. Our study provides a translational framework for interpreting neuroimaging through the lens of biomechanics and highlights the potential of virtual brain twins to inform mechanism-specific diagnosis, monitoring, and therapeutic development in TBI.

## 1. Introduction

Traumatic brain injury (TBI), caused by external forces to the head, remains a significant public health concern, impacting the life quality of >2 million Americans each year (Peterson et al., 2014) and costing the nation approximately $40 billion annually (Miller et al., 2021). TBI is not merely an acute condition but also a chronic disease with heterogeneous long-term consequences that continue to challenge the medical field. Understanding the injury mechanism is the key to reducing this societal burden with applications in producing protective headgear, developing head protection standards, designing safety systems in motor vehicles, as well as guiding early intervention after injury. Previous studies have employed experimental and computational methods to link physical loading parameters—such as acceleration, impact force, and duration—to the brain’s mechanical response, and to establish thresholds that identify loading scenarios most often associated with injury (Meaney et al., 2014). These efforts largely hinge on the prevailing hypothesis that tissue deformation—particularly axonal stretch and shear—is the principal driver of TBI pathology (Ommaya et al., 1994; Strich, 1961). However, direct evidence linking the mechanical deformation to the subsequent progressive brain pathology remains lacking.

Although this foundation of the mechanics of neurotrauma is over 80 years old (Holbourn, 1943), we still face significant challenges in merging the structural mechanical response and human pathophysiological response across the length scales. Stretch-induced functional disorder of axonal transport has been found to correlate with deformation measurements such as strain and strain rate in cultured neurons (Tang-Schomer et al., 2010) and in vivo optic nerve models (Bain and Meaney, 2000). However, the macroscale link between deformation and injury outcome is difficult to obtain in *in vivo* models because of a lack of tissue accessibility. Finite element (FE) models (FEM) have emerged as valuable tools for investigating biomechanical responses under various impact conditions (Ji et al., 2022). Tissue-level injury metrics and thresholds for predicting TBI were developed using FE reconstructions from comprehensive, multi-species TBI databases (Wu et al., 2021). In a pig TBI model, regions of large axonal deformation were spatially aligned with areas of β-amyloid precursor protein (β-APP) accumulation at 6 h post-injury, highlighting the potential of FE models to localize early axonal pathology (Hajiaghamemar and Margulies, 2021). Similar brain strain levels result in consistent injury outcomes across humans, non-human primates, and pigs (Wu et al., 2021; Hajiaghamemar et al., 2020; Wu et al., 2020), indicating a close correlation between structural mechanical response and subsequent pathophysiological responses. To solidify this linkage, however, requires high-resolution, spatially localized, and longitudinal assessments of microstructural change in the brain following TBI.

Diffusion magnetic resonance imaging (dMRI) is currently the only non-invasive method capable of mapping the axonal fiber architecture of nervous tissue, in which image contrast is based on the diffusion of water molecules in tissue (Le Bihan, 2003). As such, it has triggered tremendous hopes and expectations in detecting microstructural damage and disruptions of brain networks. dMRI-derived measures were found to be sensitive to TBI at the group level in multiple clinical studies (Crasta et al., 2022; Jia et al., 2021; Mustafi et al., 2022; Palacios et al., 2020; Wu et al., 2020). However, interpretation across studies is limited by the heterogeneity of specific outcome measures used. Due to the unknown pre-injury baseline imaging of specific patients and heterogeneity of injury scenarios in human studies, preclinical models might provide a more direct assessment of the white-matter abnormalities caused by injury. Previous dMRI studies using mouse and rat brain injury models have shown variable findings across different post-injury time points (Long et al., 2015; Nakuci et al., 2022; Zhuo et al., 2012), potentially due to factors such as injury progression, tissue recovery, and reabsorption of edema.

Combining MRI and FEM (Garimella and Kraft, 2017; Giordano and Kleiven, 2014; Wu et al., 2019) holds promise for elucidating the relationship between biomechanical forces and the spatial damage of brain tissue, offering valuable insights into the mechanisms and progression of TBI. Donat et al. (2021) demonstrated in rodent models that strain and strain rate within the first milliseconds post-injury are critical determinants of glial and axonal damage. However, the utility of small animal models is constrained by limited imaging resolution and brain size, which precludes whole-brain network analyses. Similarly, Shim et al. (2022) developed a novel pipeline in the sheep TBI model that combines FE analysis with MRI imaging for subject-specific analysis. In a follow-up study investigating the diffusional changes in the white matter tracts after TBI, Tayebi et al. utilized FE analysis to reveal that such microstructural changes would result in tissue softening and increase brain vulnerability (Tayebi et al., 2024). Despite these advances, the relationship between mechanical strain and large-scale disruptions in white matter pathways and brain network connectivity is not yet well characterized.

To address the critical knowledge gap between biomechanical deformation and white matter pathology, we leverage advanced dMRI and individualized FE simulations in large-animal (pig) models to systematically examine how mechanical strain aligns with microstructural changes across the brain, testing the hypothesis that brain pathology correlates with biomechanical deformation. Compared to previous small animal models, the pig brain—due to its larger size, white matter architecture, and gyrencephalic structure—is more predictive of human responses and clinical outcomes (Kinder et al., 2019). In this comprehensive evaluation, we utilized 36 pigs across two injury models, including 14 animals in the rotational cohort (4 sham, 10 TBI) and 22 animals in the CCI cohort (6 sham, 16 TBI), with each model implemented at two levels of severity. To the best of our knowledge, this is the first study in a large-animal model to quantitatively evaluate the correlation between biomechanical responses during injury and post-injury diffusional changes using advanced dMRI. Most importantly, this work directly tests a fundamental assumption underlying many TBI studies—that mechanical deformation leads to axonal injury—by linking strain patterns to imaging-based markers of white matter disruption. This integrative approach lays the groundwork for developing injury biomarkers grounded in both mechanical forces and neuroimaging signatures, ultimately advancing our ability to diagnose, predict, and treat TBI in human populations.

## 2. Methods

**Fig. 1.**
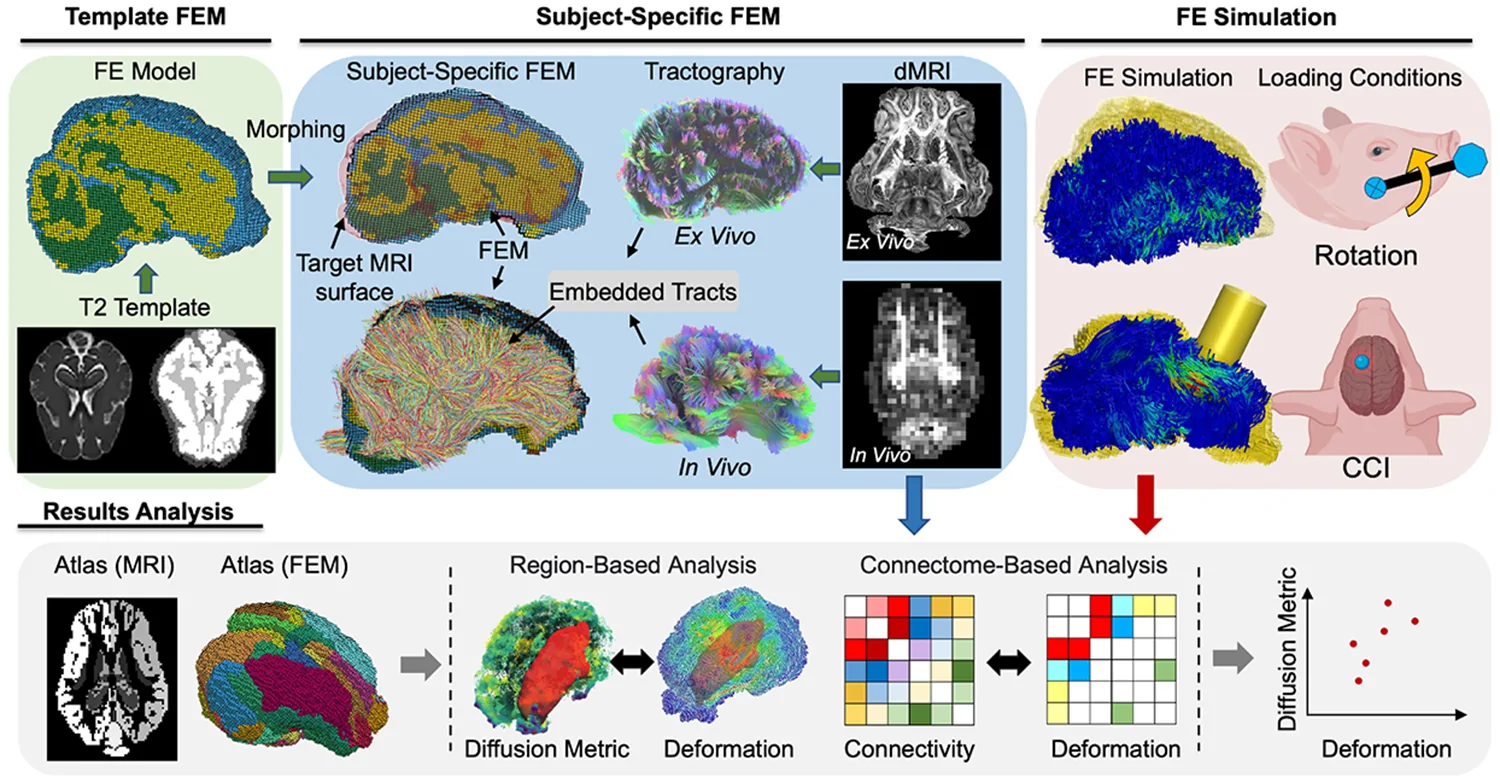
Overview of biomechanical–neuroimaging alignment framework in pig traumatic brain injury models.

Fig. 1 illustrates the workflow for comparing computationally predicted brain deformation with dMRI-derived measurements in preclinical pig TBI models. Two independent datasets were analyzed: (1) In the first dataset, pigs experienced rapid sagittal head rotation that induced either mild TBI or moderate TBI, followed by *ex vivo* dMRI of brains fixed three or fourteen days post-injury using a Bruker 9.4T scanner. (2) In the second dataset, piglets sustained CCI with a depth of depression of either 3 mm or 9 mm and underwent in vivo dMRI both before and after injury using a GE MR750 3.0T scanner. Subject-specific FE models were constructed for each animal, incorporating explicit representations of white matter fiber tracts, and were used to simulate the mechanical loading experienced during the experimental TBI. Deformation predictions from these models were compared against dMRI-derived metrics to evaluate correspondence. Additional methodological details are provided in the subsequent sections.

### 2.1. Animal experiments and MRI data acquisition

The first dataset, previously unpublished, includes fourteen 6-month-old female Yucatan pigs—four of which served as sham controls—anesthetized and subjected to rapid sagittal head rotation using a HYGE pneumatic actuator (Cullen et al., 2016). The anesthetized animal’s head was secured to a padded bite plate and mounted to the HYGE device. Head rotation in the sagittal plane occurred at mean peak angular velocities of 90–94 radians/*sec* (mildRTBI) or 108–114 radians/*sec* (modRTBI). Sham animals underwent all procedures except head rotation. Three of the modRTBI animals were euthanized three days post-injury, while the remaining three modRTBI animals, all four mildRTBI animals, and all four sham animals were euthanized fourteen days post-procedure. Their brains were extracted following transcardial perfusion with heparinized saline and 10% neutral buffered formalin. Body weight, brain weight, and gross pathology findings at extraction are summarized in Supplementary Table S1 and Fig. S1. *Ex vivo* dMRI was acquired using a spin-echo diffusion sequence (TR = 4.5 s, TE = 34 ms, 2 b = 0 images, 48 diffusion-weighted images, b = 762 s/mm^2^) in a Bruker 9.4T scanner. All experimental protocols were approved by the Institutional Animal Care and Use Committee of the University of Pennsylvania (IACUC 806,655), where these experiments were conducted.

Another dataset (Ahmed et al., 2025; Sun et al., 2024) consisted of twenty-two 8-week-old female crossbreed Landrace piglets that were subjected to a moderate-to-severe CCI using a previously published procedure (Baker et al., 2019; Kinder et al., 2019; Kinder et al., 2019). All animals were anesthetized and underwent a 20 mm craniectomy surgery at the left anterior junction of the coronal and sagittal sutures. Sixteen pigs were then secured in a CCI device, and a 15 mm impactor tip was positioned over the intact dura to induce injury in the sensorimotor network region with a depth of depression of 3 mm (CCI3) or 9 mm (CCI9). The sham group (n = 6) of pigs did not receive brain injury. All animals underwent MRI at 3 days pre-injury and 1 day, 63 days, and 119 days post-injury using a GE 32-channel fixed-site Discovery MR750 3.0 Tesla magnet. Body weight and brain size at each time point are summarized in Supplementary Table S2. In vivo dMRI was acquired using a spin-echo diffusion EPI sequence (TR = 10.0 s, TE = min-full, 3 b = 0 images, 30 diffusion weighted images, b = 1000 s/mm^2^). All animals were sedated using the same protocol as craniectomy surgery and maintained with mild anesthesia using isoflurane in oxygen (1.5%, Abbot Laboratories) for the duration of the scan. This study was performed in accordance with the National Institutes of Health (NIH) Guide for the Care and Use of Laboratory Animals guidelines. The animal study protocol was reviewed and approved by the Institutional Review Board of the University of Georgia (Animal Use Protocol A2023 07–021-A13).

A summary of both datasets is provided in Table 1.

### 2.2. Image analysis

dMRI in Digital Imaging and Communications in Medicine (DICOM) format was converted into Neuroimaging Informatics Technology Initiative (NIfTI) format using the “dcm2niix” software (MRIcroGL v1.2.20220720). Non-brain parts were removed using the Brain Extraction Tool (BET) in the FMRIB Software Library (FSL). dMRI data were reconstructed in native space in DSI Studio (http://dsi-studio.labsolver.org) using Generalized Q-sampling Imaging (GQI) (Yeh et al., 2010) with a diffusion sampling length ratio of 0.6 for *ex vivo* and 1.25 for in vivo scans. For *ex vivo* DWI, bias field correction was performed prior to reconstruction due to the presence of global intensity inhomogeneity. dMRI preprocessing generated both conventional diffusion tensor imaging (DTI) metrics—fractional anisotropy (FA), radial diffusivity (RD), mean diffusivity (MD), and axial diffusivity (AD)—and the GQI-derived metric, quantitative anisotropy (QA). Deterministic whole-brain fiber tracking was conducted by seeding 1000,000 streamlines, employing augmented tracking strategies to enhance reproducibility.

To enable spatial comparison across animals and time points (Section 2.5, Data Analysis), a porcine brain parcellation atlas (Saikali et al., 2010) was nonlinearly registered to each subject’s image space using the nonlinear registration algorithm implemented in DSI Studio. For the rotational injury dataset (*ex vivo*), the atlas was registered to the B0 image (0.5 mm isotropic resolution), whereas for the CCI dataset (in vivo), the atlas was registered to the B0 image acquired at 2 mm isotropic resolution. The atlas regions of interest (ROIs) were divided into three anatomical groups: cerebral cortex (CTX), subcortical cerebrum (SCB), and brainstem & cerebellum (BS-CBLM), as detailed in Supplementary Table S3. The cortical ROIs (54 regions, Table S3) were used to define regions for generating structural connectivity (SC) matrices by filtering streamlines that traversed between cortical regions. Connectivity between ROI pairs was quantified by normalizing the number of connecting streamlines or by sampling along tracts to compute mean dMRI metrics (FA, MD, RD, AD, QA). All CTX, SCB, and BS-CBLM ROIs were used for generating region-specific diffusion metrics. To ensure robustness of regional estimates and account for differences in spatial resolution between datasets, ROIs containing fewer than 10 voxels in the in vivo space (lowest spatial resolution) were excluded from downstream analyses.

### 2.3. Development and validation of FEM

We created a high-fidelity FE model with segmented brain parenchyma and cerebrospinal fluid (CSF) from a Yucatan pig T2-weighted MRI brain template (Chang et al., 2020). The MRI template (0.5 × 0.5 × 0.5 mm) was first downsampled to a 1 mm^3^ voxel size. Each 1 mm isotropic voxel in the image was directly converted into a corresponding 1 mm hexahedral element and categorized based on its segmented tissue type using a custom MATLAB script (Giudice et al., 2019). Quadrilateral shell elements were created on the external surface of the CSF layer to represent the dura/skull boundary and were made rigid. All interfaces were continuous and connected through shared nodes.

Subject-specific models, constructed to represent the brain anatomies of the experimental animals, were generated using a non-linear morphing technique (Giudice et al., 2020), which leverages image registration transformations to morph the template FE model to align with the individual brain (Supplementary Fig. S2). A FE mesh of white matter (axonal) fibers was explicitly embedded into the solid mesh of each subject-specific model using a modeling technique previously described (Wu et al., 2019). The fiber mesh was generated directly in the native space from a downsampled tractography reconstructed from dMRI using a custom MATLAB script. Downsampling was conducted by removing repeated tracks within certain distances, resulting in approximately 10,000 tracks. The fiber mesh was in the same anatomical space as the volumetric subject-specific brain mesh; they were constrained as embedded elements using the *CONSTRAINED_BEAM_IN_SOLID Keyword in ANSYS LS-DYNA, in which both accelerations and velocities of the fibers were constrained by the surrounding volumetric mesh. On average, 58.0 ± 5.7% of parenchymal elements contained embedded axonal elements, indicating substantial spatial coverage of the reconstructed white matter network.

In this study, gray and white matter were modeled with the same hyper-viscoelastic constitutive model. The anisotropy and heterogeneity of material properties were introduced through the embedded fiber architecture, modeled using elastic beam elements. Consistent with previous work (Wu et al., 2019), the inclusion of fibers has a limited effect on global strain metrics while contributing to localized variations in strain distributions (Supplementary Fig. S3). The material properties for the CSF (peripheral and internal) were adapted from previous brain models (Giudice et al., 2021) and modeled using a linear viscoelastic material with very low stiffness (G_0_ = 0.5 kPa; G_∞_ = 0.1 kPa). The selection of the numerical implementation approach was informed by Giudice et al. (2019b). The details on material properties are summarized in the Supplementary Table S4.

The hyper-viscoelastic constitutive model used for brain parenchyma (white and gray) is a one-term Ogden quasi-linear viscoelastic (QLV) model with five-term Prony series (Eq. (1)-(3)

(1) $$W=\frac{\mu }{\alpha }(\lambda_{1}^{\alpha }+\lambda_{2}^{\alpha }+\lambda_{3}^{\alpha }-3)$$

(2) $$\sigma =\int_{0\;}^{\;t}g(t-\tau )\frac{\partial \sigma_{e}}{\partial \tau }d\tau$$

(3) $$g(t)=g_{\infty }+\sum_{i=1}^{5}g_{i}e^{-t∕\tau_{i}}$$

In the strain energy density function (*W*), *λ*_1_, *λ*_2_, and *λ*_3_ are the three principal stretches, *μ* is the shear modulus, and *α* is a unitless nonlinearity coefficient. The infinitesimal shear modulus (i.e., initial slope of the non-linear shear stress-strain curve), *μ*_0_, can be obtained as a function of the material parameters $\mu_{0}=\frac{1}{2}\mu \alpha$. The stress response is a function of the instantaneous elastic response (*σ_e_*) due to the Ogden model and the time-dependent relaxation behavior governed by the Prony series *g*(*t*), where *g*_∞_ is the long-term equilibrium modulus fraction, and *gi* are the coefficients at relaxation times *τ_i_*. The sum of *g*_∞_ and *g_i_* is equal to 1.

**Fig. 2.**
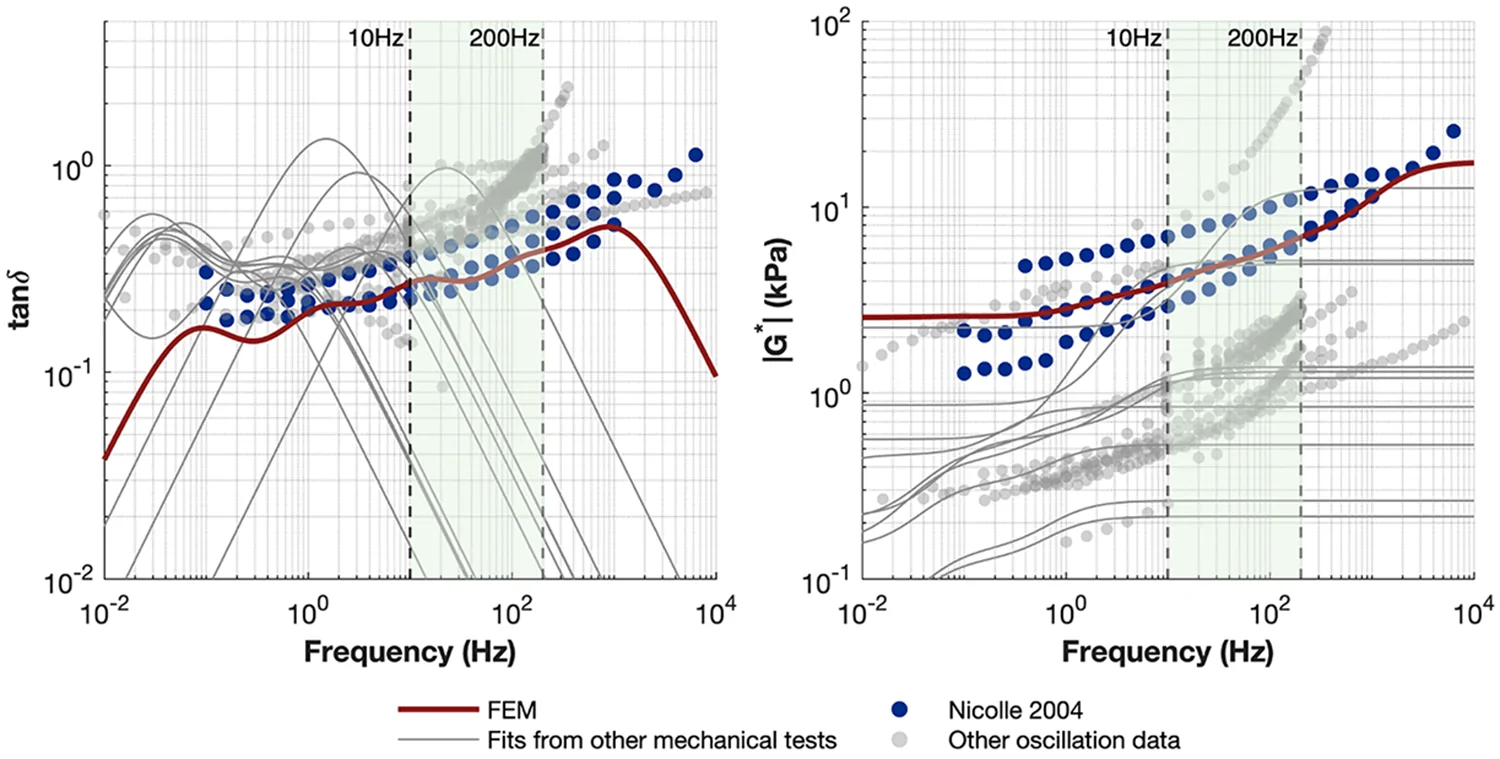
Comparison of loss tangent (left) and complex modulus (right) between the FEM brain material model and Nicolle’s experimental data. Additional oscillatory data were obtained from shear tests on porcine brain tissue reported in the literature. Overlaid fits derived from other mechanical tests were calculated to enable comparison with oscillatory results. The shaded region (10–200 Hz) highlights the frequency range most relevant to the dynamic loading conditions observed in the TBI models studied.

The material parameters for the brain tissue in FEM were calibrated against experimental data from Nicolle et al. 2004 (Nicolle et al., 2004), which characterized the frequency-dependent viscoelastic behavior of porcine brain tissue over a broad range. Fig. 2 presents the fitted complex modulus and loss tangent (tan*δ*), compared against Nicolle’s experimental data and overlaid with additional porcine brain mechanical data reported in the literature (Nicolle et al., 2004; Arbogast et al., 1997; Bilston et al., 2001; Brands et al., 1999; Fallenstein et al., 1969; Garo et al., 2007; Hrapko et al., 2006; Peters et al., 1997; Shen et al., 2006; Shuck and Advani, 1972; Thibault and Margulies, 1998) and fits derived from other mechanical tests of porcine brain (Miller and Chinzei, 2002; Prange and Margulies, 2002; Rashid et al., 2014; Rashid et al., 2013; Rashid et al., 2012; Sundaresh et al., 2021).

**Fig. 3.**
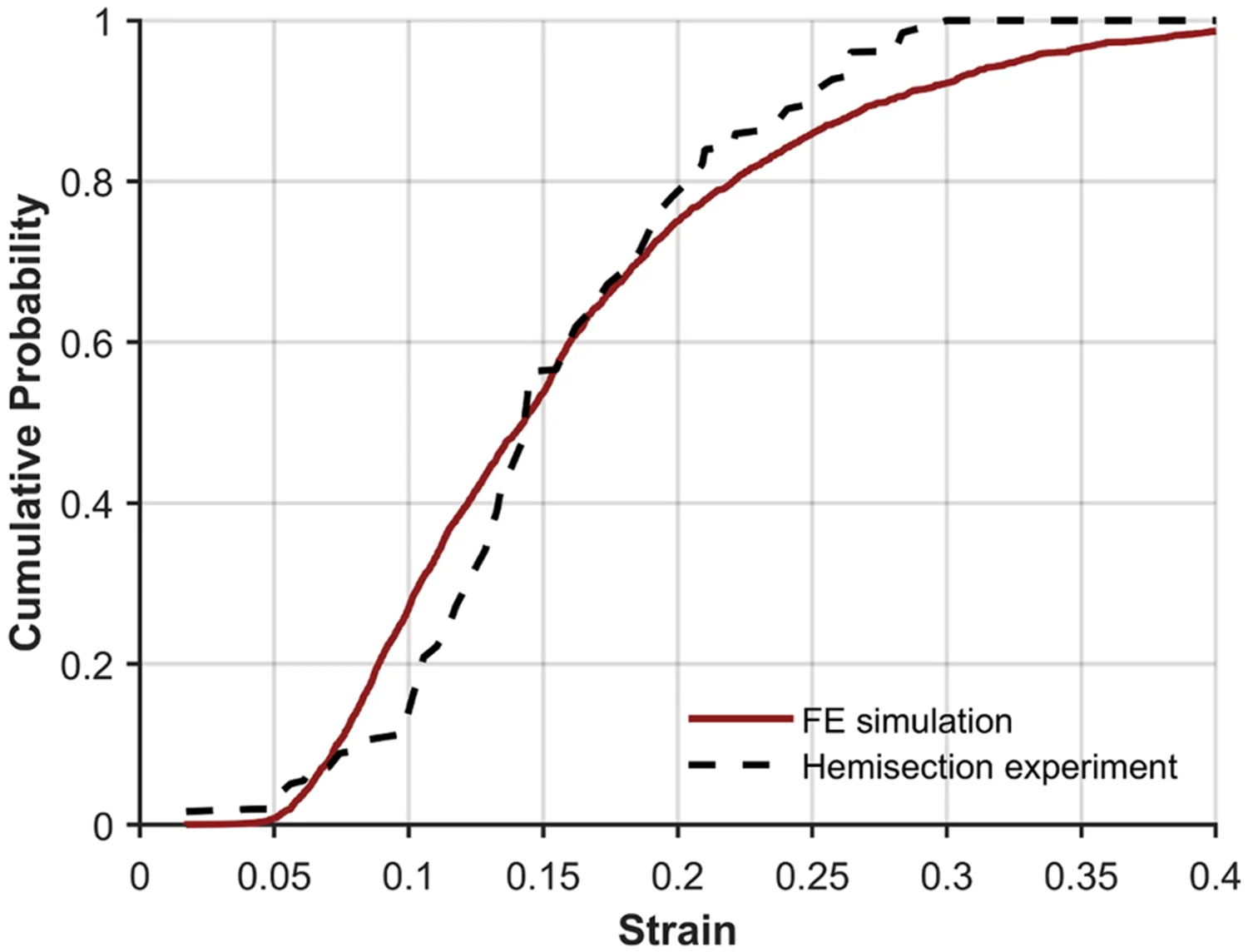
Surface strain distribution from the FEM hemisection simulation and the ex vivo hemisection experiment.

Model validation was performed using deformation measurements from an *ex vivo* hemisection experiment (Hajiaghamemar et al., 2020; Ibrahim et al., 2010; Sullivan et al., 2015). In this experiment, a transected pig head was subjected to 65-degree axial-plane rotation at 50 rad/s, and ink markers placed on the exposed brain surface were tracked with a high-speed digital camera to capture tissue motion. To replicate this setup, we developed a hemisection brain FEM with comparable geometry and applied the experimentally measured rotational velocity trace as the model’s boundary condition. The simulated deformation showed good agreement with experimental measurements (Fig. 3) using the calibrated viscoelastic material properties, without requiring further parameter tuning.

### 2.4. Simulation of animal experiments

We performed parallel, non-linear, explicit simulations using ANSYS LS-DYNA (R13.1.1–17, double precision) with subject-specific loading conditions. The non-impact rotational tests were simulated by prescribing experimentally measured sagittal-plane rotational velocity profiles to the FE brain model through a rigid dura. The CCI simulations were performed by applying the experimentally recorded time-displacement curves to a rigid impactor tip that was typically centered over the left motor cortex. The impact location was confirmed using structural MRI, where the craniectomy site was clearly visible. CCI simulations (3 mm and 9 mm impact depths) were run for 40 ms, and rotational simulations were run for 60 ms, encompassing the primary deformation and subsequent dynamic response. Sham animals were not simulated because no mechanical loading or injury was applied.

Mechanical deformation was quantified using two complementary strain measures: element-wise maximum principal strain (MPS) for the solid brain tissue, and element-wise maximum axial strain (MAS) along axonal fiber tracts. To enable region-specific analysis, the same cortical parcellation atlas used for dMRI analysis in DSI Studio was applied to the FEM (Supplementary Fig. S5). For each brain ROI, MPS values from all elements within the ROI were extracted, and the 95th percentile was recorded to yield a single representative deformation metric per region per subject. Similarly, MAS values were sampled along the fiber tracts connecting pairs of cortical ROIs. For each pairwise connection, the 95th percentile of MAS was computed across the sampled tracts, resulting in a subject-specific damage connectome that characterizes inter-regional axonal strain exposure during injury.

### 2.5. Data analysis

For the CCI cases with in vivo dMRI data, group-wise comparisons of dMRI metrics were conducted using two-sample *t*-tests. Region-wise comparisons across different pre- and post-injury time points were also performed using two-sample *t*-tests. Changes in dMRI metrics were quantified within subjects by subtracting each animal’s pre-injury region-based values and connectivity measures from the corresponding post-injury measurements. Resulting p-values were corrected using the Bonferroni method.

For the rotational injury cases with *ex vivo* dMRI data, similar statistical analyses were applied. Group-wise comparisons of dMRI metrics and region-wise comparisons across time points were performed using two-sample *t*-tests. As individual pre-injury scans were not available in this cohort, changes in dMRI metrics were estimated by comparing each TBI subject to the four sham animals, which served as a pooled baseline reference. P-values were not corrected to highlight potential spatial and temporal trends, given the smaller sample size compared to the CCI dataset.

In addition to region-wise analyses, edgewise comparisons were performed on the connectivity matrices. Here, edges refer to connections between pairs of ROIs in the connectivity matrices, defined either by the normalized number of connecting streamlines (fiber count) or by mean dMRI metrics (FA, MD, RD, AD, QA) sampled along the fiber tracts. Group-wise differences between TBI and baseline animals (sham animals in the rotational cohort or pre-injury scans in the CCI cohort) were evaluated using two-sample *t*-tests at each edge.

We performed linear regression analysis at both the region-wise and edgewise levels to examine the relationship between mechanical deformation and dMRI alterations. For each subject, the percentage change in dMRI metrics was regressed against the predicted deformation metrics. Pearson correlation coefficients (r) were computed to quantify the strength of these associations. To evaluate the statistical significance of TBI-induced correlations, r-values from injured-subject comparisons were compared to sham-reference correlations derived using the same TBI deformation fields and pairwise sham MRI differences. Statistical comparisons were performed using two-tailed *t*-tests, stratified by injury severity and post-injury time point. We additionally computed voxel-wise mean and standard deviation maps of diffusion and strain metrics to characterize inter-subject variability and group-level spatial patterns. Lastly, for the most significant associations, linear relationships between deformation and dMRI metrics were characterized by pooling data across relevant subjects.

## 3. Results

### 3.1. Moderate rotational TBI induces early deep-brain and delayed cortical and subcortical diffusion abnormalities

**Fig. 4.**
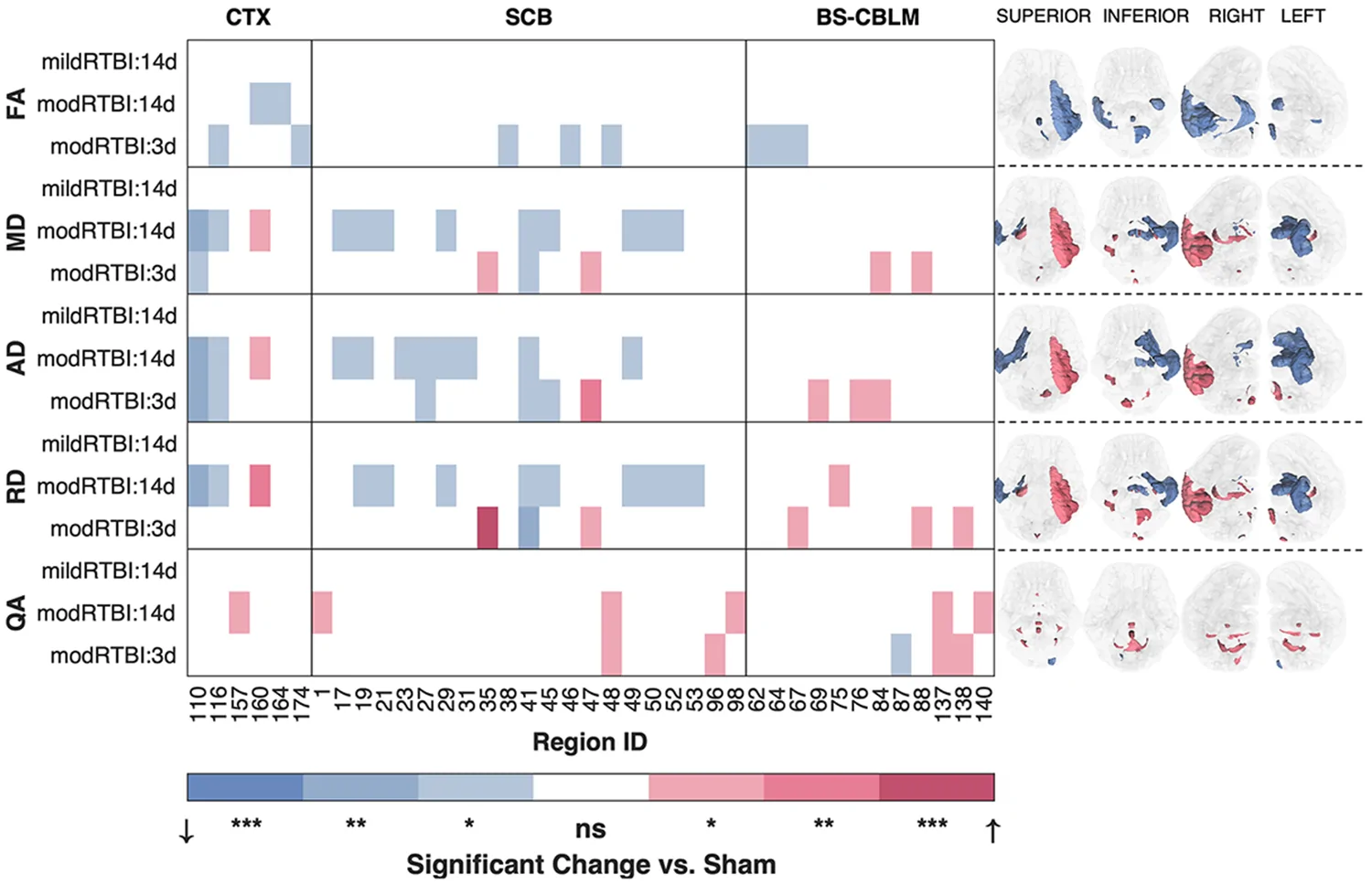
Regional changes in dMRI metrics following rotational TBI. Heatmaps (left) and 3D brain overlays (right) illustrate significant changes in five metrics—fractional anisotropy (FA), mean diffusivity (MD), axial diffusivity (AD), radial diffusivity (RD), and quantitative anisotropy (QA)—across cortical (CTX), subcortical (SCB), and brainstem-cerebellar (BS-CBLM) regions. Each row corresponds to a TBI group (mildRTBI or modRTBI) at either 3 or 14 days post-injury. Color indicates the direction of change relative to sham animals (red: increase; blue: decrease), while color intensity reflects statistical significance (*p < 0.05, **p < 0.01, ***p < 0.001; uncorrected). Group-wise comparisons were performed using two-sample *t*-tests, and only regions showing statistically significant changes from the sham group are displayed.

Group-wise comparisons of region-specific dMRI metrics revealed injury severity- and time-dependent effects following rotational TBI (Fig. 4). No statistically significant changes were detected in the mildRTBI group at 14 days post-injury. In contrast, animals in the modRTBI group exhibited notable diffusion alterations, particularly in subcortical and brainstem-cerebellar regions. At 3 days post-injury, changes were primarily localized to deep brain structures, with widespread increases in MD, AD, and RD, accompanied by reductions in FA. QA was elevated, especially in inferior brain regions. By 14 days post-injury, diffusion abnormalities expanded into both subcortical and cortical regions, with reductions in MD, RD, and AD. FA showed minimal abnormality at 14 days post-injury in the modRTBI group. Notably, the apparent asymmetry is primarily observed in DTI-derived metrics (FA, MD, AD, RD), whereas QA exhibits greater left–right symmetry.

We also performed edgewise comparisons of SC matrices (see individual SC matrices in Supplementary Fig. S5). Only a small subset of edges (2.5%) showed statistically significant differences (p < 0.05, uncorrected). Among the various SC metrics, the matrix defined by streamline counts exhibited the greatest number of significant edges (Supplementary Fig. S6).

### 3.2. CCI induces delayed and spatially propagating diffusion and connectivity changes from the impact site

**Fig. 5.**
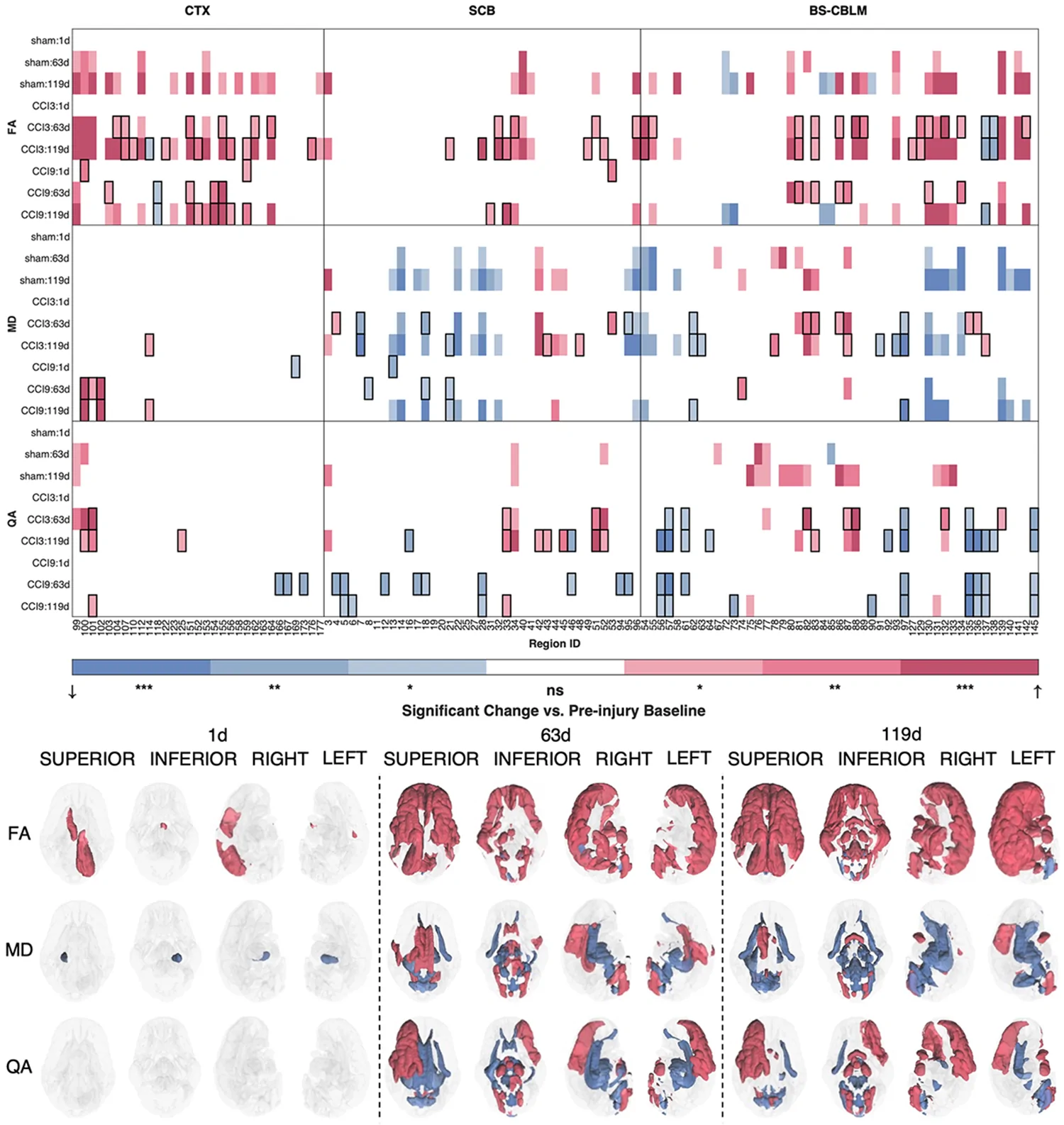
Regional changes in diffusion MRI metrics following controlled cortical impact (CCI). Heatmaps (top) and 3D overlays (bottom) show statistically significant changes in fractional anisotropy (FA), mean diffusivity (MD), and quantitative anisotropy (QA) across cortical (CTX), subcortical (SCB), and brainstem-cerebellar (BS-CBLM) regions. Each row corresponds to a specific group and time point, including sham animals and animals with CCI3 or CCI9 at 1, 63, or 119 days post-injury. Colors indicate the direction of change relative to each animal’s own pre-injury baseline scan (red: increase; blue: decrease), with color intensity denoting statistical significance (*p < 0.05, **p < 0.01, ***p < 0.001; corrected). Regions that show significant changes in TBI groups but not in sham groups are outlined. Group-wise comparisons were performed using *t*-tests, and only regions showing statistically significant changes are displayed. Note that sham animals underwent craniectomy without cortical impact.

Longitudinal analysis of dMRI metrics revealed delayed and spatially widespread microstructural alterations following CCI, with patterns varying by injury severity and time post-injury (Fig. 5). Sham animals exhibited minimal longitudinal changes, particularly in QA, supporting the reliability of baseline scans.

In both CCI3 and CCI9 groups, most diffusion changes were not evident at 1 day post-injury but emerged more clearly at later time points. At 63 and 119 days post-injury, widespread changes were observed, including notable increases in FA—a pattern that is counterintuitive, but these FA increases were also pronounced in the sham group, suggesting a non-injury-related effect, potentially reflecting ongoing white matter maturation. Changes in MD and QA varied by region, with both increases and decreases observed, though increases were especially pronounced near and at the impact site. QA appeared to be a more stable metric over time, with fewer significant changes detected in the sham group and more localized alterations in the TBI groups compared to FA. AD and RD were not prominently featured, as they closely followed the trends observed in MD. Statistically significant regional differences were determined using Bonferroni-corrected paired *t*-tests comparing each post-injury time point to the animal’s pre-injury baseline. Outlined regions in Fig. 5 highlight those showing significant changes in the TBI group but not in shams.

Edgewise comparisons of SC matrices revealed more pronounced alterations in the CCI model than in the rotational injury dataset (see individual SC matrices in Supplementary Fig. S7). Among the various SC definitions, matrices based on streamline count and QA were the most stable, showing minimal longitudinal changes in the sham group. In animals with CCI9, QA-based SC differences emerged as early as 1 day post-injury and persisted through later time points, indicating early disruption of network-level microstructure. In contrast, CCI3 animals exhibited detectable SC changes only at the chronic stage (119 days post-injury), suggesting a delayed and milder pattern of network alteration (see Supplementary Fig. S8).

### 3.3. Predicted brain deformation correlates with early diffusion and connectivity changes after moderate rotational TBI

To investigate the link between mechanical deformation and observed MRI alterations, we performed linear regression analyses between predicted regional deformation metrics and percentage changes in diffusion and SC measures. Pearson correlation coefficients (r) were computed for each subject and grouped by post-injury time point and injury severity (mildRTBI vs. modRTBI).

**Fig. 6.**
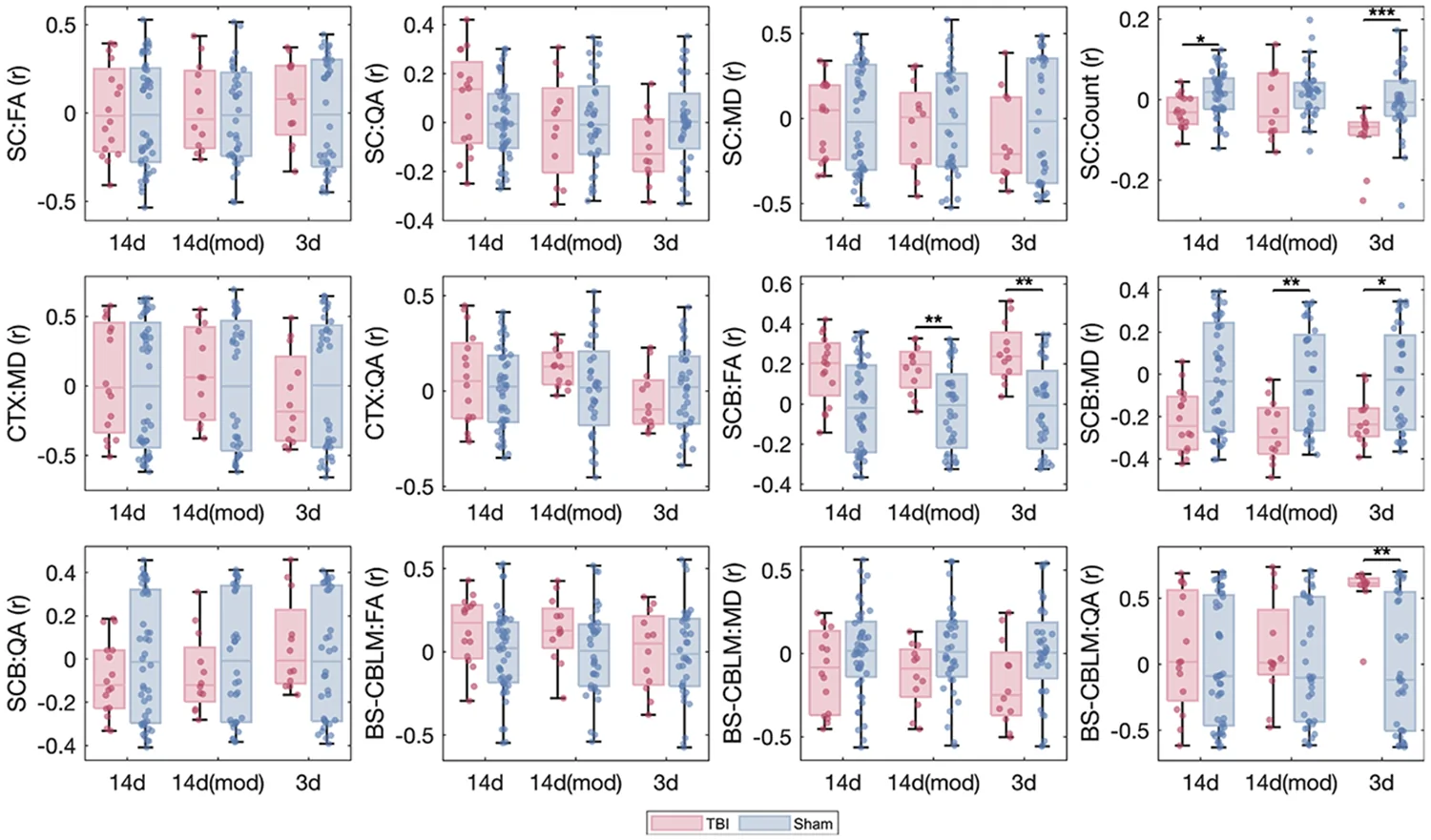
Correlation between biomechanical deformation and diffusion/connectivity changes across brain regions. Boxplots show the distribution of subject-wise Pearson correlation coefficients (r) between predicted biomechanical deformation metrics (MPS or MAS) and percentage changes in diffusion or structural connectivity (SC) measures. Results are grouped by brain region (CTX: cortex, SCB: subcortical, BS-CBLM: brainstem-cerebellum), imaging metric (FA, MD, QA), and time point (3 days for modRTBI, 14 days for mildRTBI, or 14 days for modRTBI subgroup). SC matrices were defined using FA, QA, MD, and streamline count. Statistically significant differences between mildRTBI and modRTBI groups are marked with asterisks (*p < 0.05, **p < 0.01, ***p < 0.001; uncorrected, two-sample *t*-tests). Red and blue boxes indicate TBI and sham groups, respectively. Each red dot represents a correlation estimate derived from the deformation field of one TBI subject paired with diffusion/connectivity changes computed between the injured subject and one sham reference. Blue dots represent sham-reference correlations derived from the same TBI deformation field and diffusion/connectivity differences between two sham references. A positive correlation indicates that greater predicted deformation is associated with increases in the diffusion metric after injury, whereas a negative correlation implies a decrease in the metric with greater deformation.

As shown in Fig. 6, several regional and edge-level associations reached statistical significance. In the subcortical (SCB) region, significant positive correlations were observed between MPS and both FA and MD changes at 14 days post-injury (p < 0.01). BS-CBLM QA changes showed significant positive correlations with deformation (r = 0.573) in the modRTBI group. At the network level, structural connectivity based on streamline counts showed a significant negative correlation with MAS at 3 days in the modRTBI group (p < 0.001) and at 14 days in the mildRTBI group (p < 0.05), suggesting biomechanical loading may drive measurable changes in connection strength.

Overall, stronger correlations were observed in acute time points (3 days) compared to later phase (14 days), and in modRTBI cases more so than mildRTBI. These findings suggest that spatial deformation patterns are associated with both regional and network-level alterations, particularly in subcortical and brainstem regions.

**Fig. 7.**
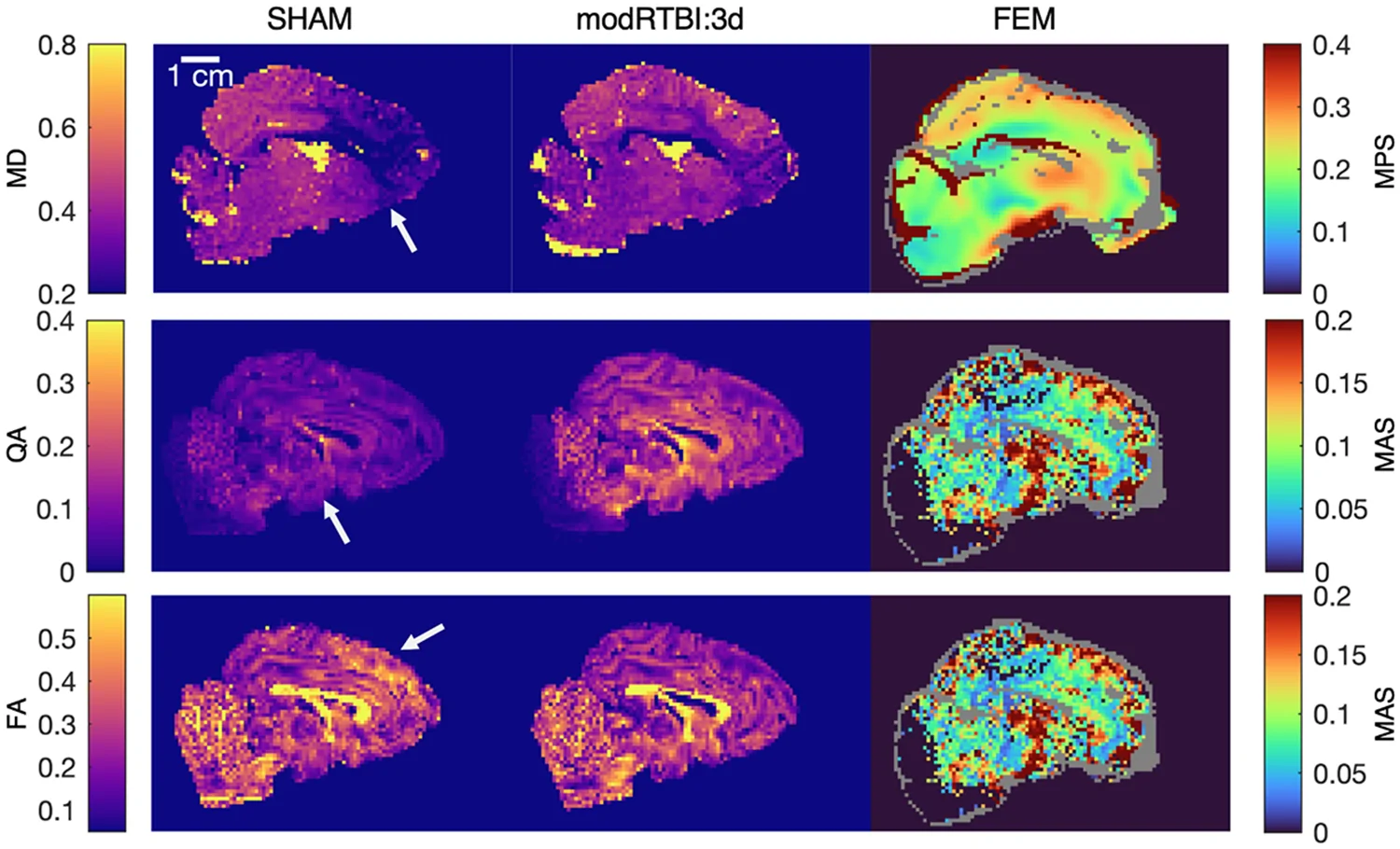
Spatial correspondence between diffusion metric alterations and predicted biomechanical deformation in moderate rotational TBI. Voxel-wise maps of mean diffusivity (MD), quantitative anisotropy (QA), and fractional anisotropy (FA) are shown for a representative sham animal (left column) and a moderate TBI (modRTBI) subject at 3 days post-injury (middle column). The right column shows the corresponding finite element predictions of mechanical strain: maximum principal strain (MPS) for MD and FA, and maximum axonal strain (MAS) for QA and FA. Regions with elevated MPS and MAS show spatial alignment with areas of altered diffusion metrics in the injured brain, particularly in deep white matter and periventricular regions. White arrows highlight representative locations of spatial correspondence across modalities. For visualization, MAS values were projected onto the nearest voxels of the 3D imaging grid from the corresponding finite element mesh elements. All diffusion and deformation maps are displayed in template space for spatial correspondence.

To further examine the spatial relationship between predicted biomechanical deformation and diffusion alterations, we visualized voxel-wise maps of MD, QA, and FA from a representative modRTBI subject alongside corresponding finite element predictions of MPS and MAS at 3 days post-injury (Fig. 7). Regions of elevated MPS and MAS were observed to spatially coincide with areas showing abnormal diffusion metrics, particularly in deep white matter and periventricular regions. This visual correspondence supports the hypothesis that localized mechanical strain contributes to subsequent microstructural disruption. While MD and FA maps exhibited noticeable spatial inhomogeneity, limiting their reliability for voxel-wise comparisons, QA and tractography-based metrics such as streamline count appeared more spatially stable.

### 3.4. Mechanical deformation predicts acute-to-chronic diffusion changes in severe CCI

Similarly, we performed linear regression analyses for the CCI cases to compare predicted deformation metrics and percentage changes in dMRI-derived measures. Pearson correlation coefficients (r) were computed for each subject and grouped by post-injury time point and injury severity (CCI3 vs. CCI9).

**Fig. 8.**
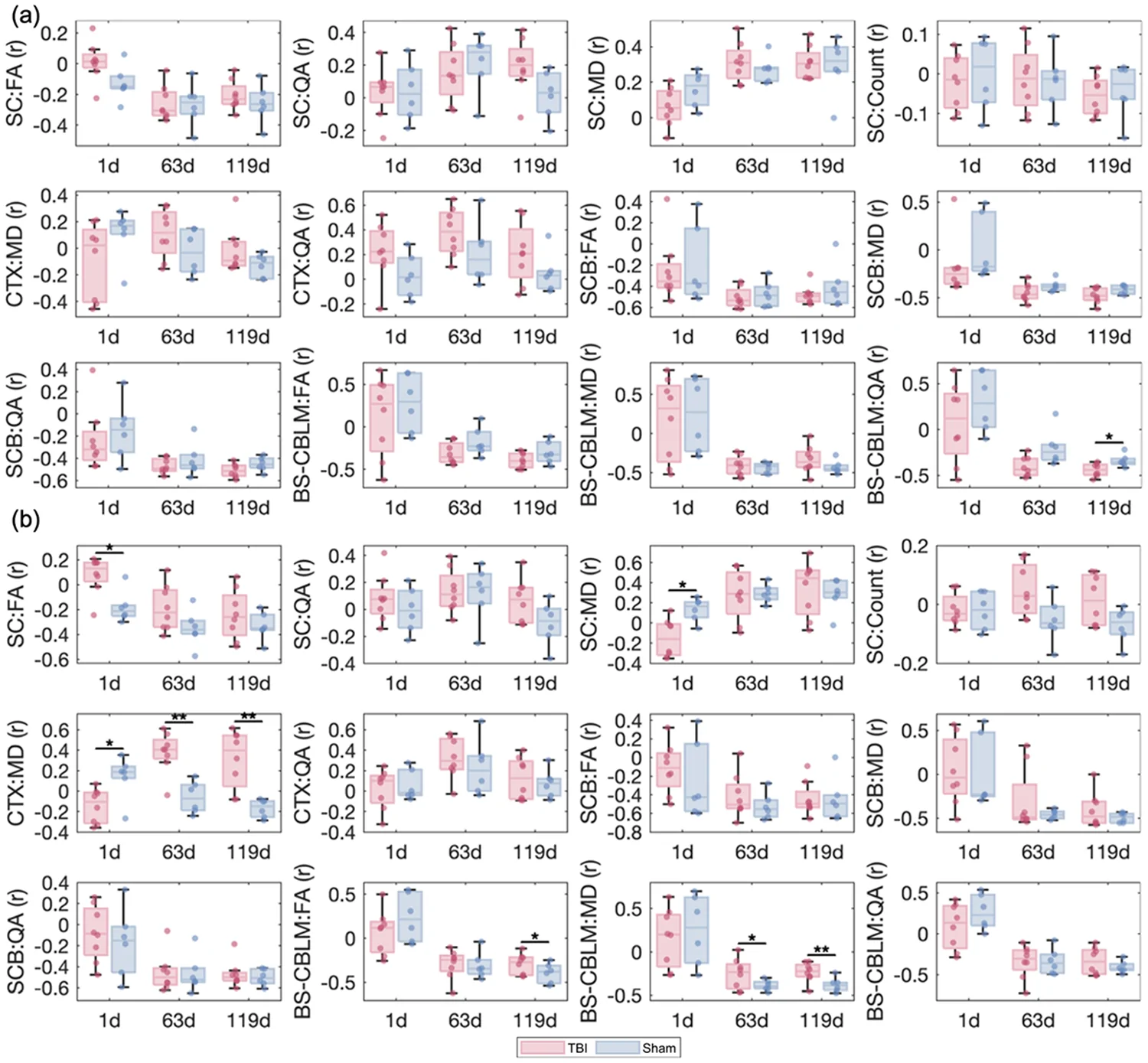
Correlation between biomechanical deformation and diffusion/connectivity changes in the CCI model. (a) CCI3; (b) CCI9. Boxplots show the distribution of subject-wise Pearson correlation coefficients (r) between predicted deformation metrics (MPS or MAS) and percentage changes in regional diffusion metrics and structural connectivity (SC) measures. Results are grouped by time point (1, 63, and 119 days post-injury) and by group (TBI in red, sham in blue). Correlation values are shown for cortex (CTX), subcortical white matter (SCB), and brainstem-cerebellar (BS-CBLM) regions across different imaging features: FA, MD, QA, and SC matrices defined by FA, QA, MD, and streamline count. Asterisks indicate statistically significant differences between TBI and sham groups (p < 0.05, *p < 0.01, **p < 0.001; uncorrected, two-sample t-test). Each red dot represents a correlation estimate derived from the deformation field of one TBI subject paired with diffusion/connectivity changes computed between the injured subject and its pre-injury reference. Blue dots represent sham-reference correlations derived from the same TBI deformation field and diffusion/connectivity differences between sham subjects and their corresponding pre-injury references. A positive correlation indicates that greater predicted deformation is associated with increases in the diffusion metric after injury, whereas a negative correlation implies a decrease in the metric with greater deformation.

As shown in Fig. 8, significant correlations were limited in CCI3, with the exception of QA in the brainstem-cerebellar (BS-CBLM) region at 63 and 119 days. In contrast, several significant associations were observed in the CCI9 group. Notably, MD in the cortex (CTX) showed consistent, significant correlations with MPS across all time points (1, 63, and 119 days), reflecting the direct mechanical loading at the cortical impact site.

SC matrices based on FA and MD also showed significant correlations with deformation in the CCI9 group. Interestingly, SC FA initially showed a positive correlation at 1 day post-injury—likely reflecting early post-impact signal increase—but these effects diminished over time. In the BS-CBLM region, MD was significantly correlated with deformation at both 63 and 119 days, highlighting persistent vulnerability in deep structures remote from the impact site.

Among all metrics, MD at the cortical impact site exhibited the clearest spatiotemporal trend, demonstrating a decreased MD (an increased FA) at the site of injury at 1day post-injury which reversed by increasing at later time points—while maintaining strong correlation with predicted strain. This suggests that MD may be a sensitive and temporally dynamic marker of tissue response to focal mechanical insult in CCI. Collectively, these findings support a region-specific, deformation-driven pattern of injury, with the strongest and most consistent effects observed in cortical MD and BS-CBLM measures in the CCI9 group.

**Fig. 9.**
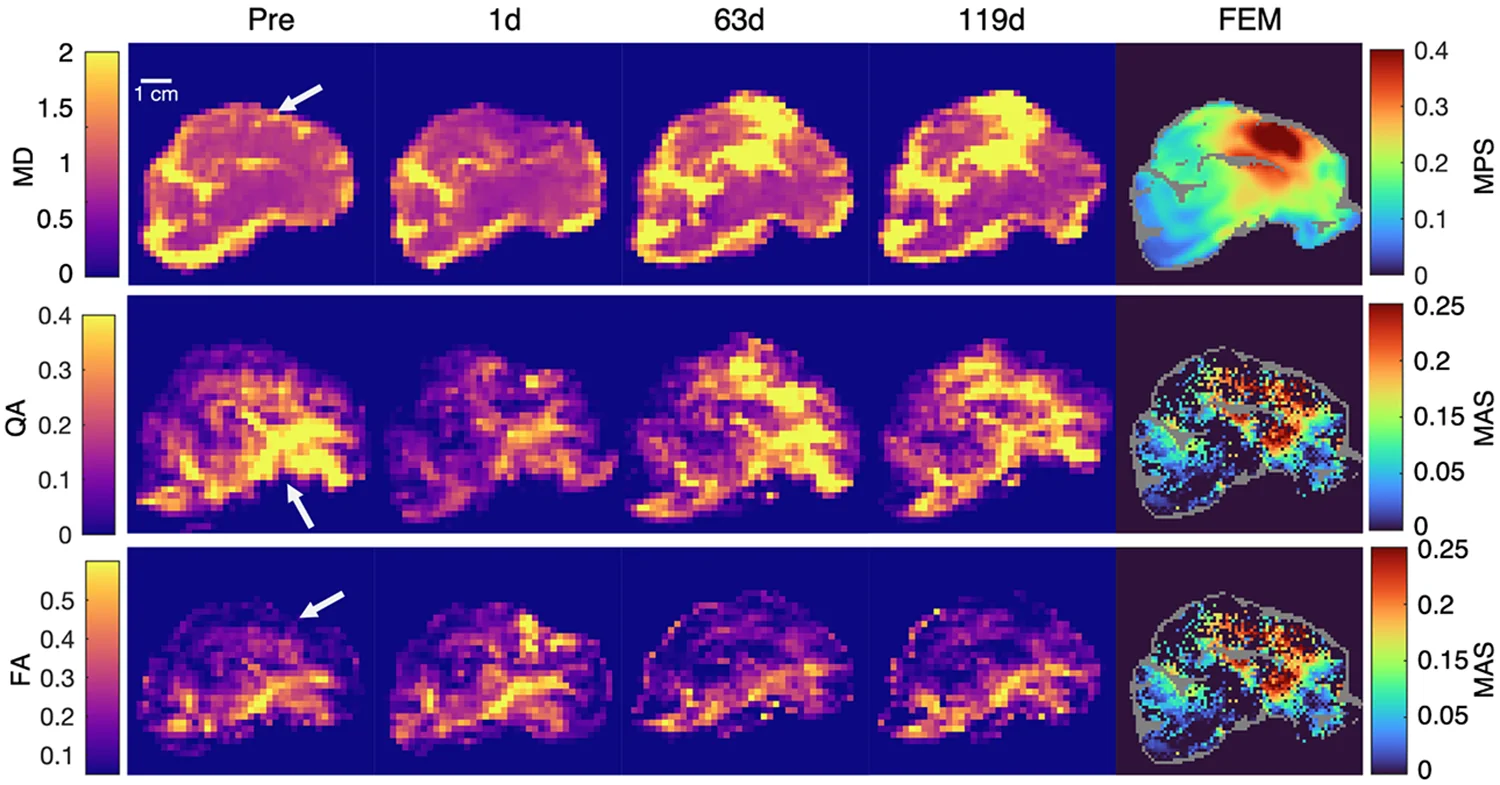
Longitudinal evolution of diffusion metric changes and spatial correspondence with predicted biomechanical deformation in a representative CCI9 subject. Voxel-wise maps of mean diffusivity (MD), quantitative anisotropy (QA), and fractional anisotropy (FA) are shown across four time points: pre-injury, and 1, 63, and 119 days post-injury. The rightmost columns show predicted maximum principal strain (MPS) and maximum axonal strain (MAS) from the subject-specific finite element model. Spatially extensive deformation was observed near the impact site, as indicated by elevated MPS and MAS values. These regions of elevated strain corresponded with gradual and spatially spreading increases in MD and decreases in QA and FA over time. For visualization, MAS was projected onto the nearest voxels of the imaging grid. All diffusion and deformation maps are displayed in template space for spatial correspondence.

To further visualize this relationship, Fig. 9 presents longitudinal voxel-wise diffusion maps (MD, QA, FA) from a representative CCI subject alongside predicted deformation maps (MPS and MAS). While diffusion maps showed little change at 1 day post-injury, widespread increases in MD and decreases in QA and FA emerged at 63 and 119 days. These changes were spatially aligned with regions of elevated MPS and MAS near the impact site, suggesting a spatial correspondence between early mechanical insult and delayed degeneration.

### 3.5. Spatial variability analysis reveals consistent group-level patterns despite inter-subject differences

**Fig. 10.**
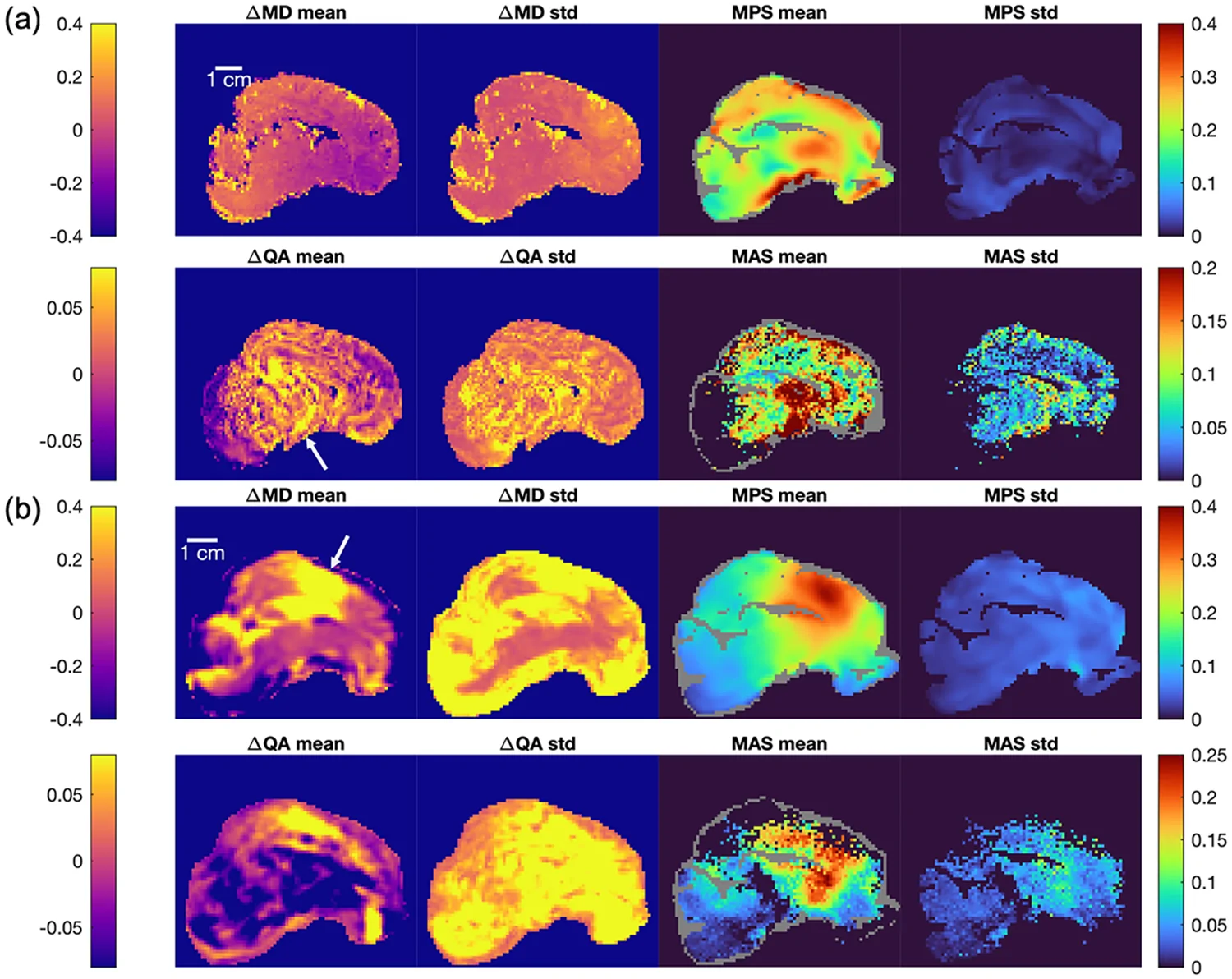
Spatial variability of diffusion and strain metrics across animals. (a) moderate TBI (modRTBI) subject at 3 days post-injury (b) severe CCI (CCI9) at 119 days post-injury. Voxel-wise mean and standard deviation maps are shown for diffusion metrics (ΔMD, ΔQA) and strain measures (MPS, MAS). Δ maps represent absolute changes relative to baseline (post–pre for CCI; subject–sham mean for rotational injury). The same color scale is used for mean and standard deviation maps to facilitate direct comparison of signal magnitude and variability.

To further interpret the relationship between diffusion changes and mechanical deformation, we examined the spatial variability of diffusion and strain metrics across subjects. Results are shown for the modRTBI cohort at 3 days post-injury (Fig. 10a) and the CCI9 cohort at 119 days post-injury (Fig. 10b); additional cohorts are provided in Supplementary Fig. S9. Despite notable inter-subject variability, particularly in diffusion metrics, the spatial patterns of injury-related changes remain evident in the group mean and are not washed out by averaging. In the rotational model, consistent patterns are observed in deep brain regions at the base of the brain in ΔQA, while in the CCI model, elevated ΔMD is localized to the cortical impact region.

In contrast to diffusion metrics, strain measures (MPS and MAS) exhibit lower variability and greater spatial consistency across subjects. This suggests that variability in diffusion metrics cannot be fully explained by differences in brain morphology, white matter architecture, or loading conditions, which were accounted for in the subject-specific FE modeling framework. The observed inter-subject variability in diffusion metrics likely reflects a combination of subject-specific biological responses, particularly edema and other acute physiological effects in the in vivo CCI cohort, and variability arising from underlying tissue structure and injury-related microstructural differences in the *ex vivo* rotational cohort, where fixation reduces physiological confounds.

### 3.6. Consistent mechanistic relationship observed across TBI models despite differences in strain-diffusion manifestation

**Fig. 11.**
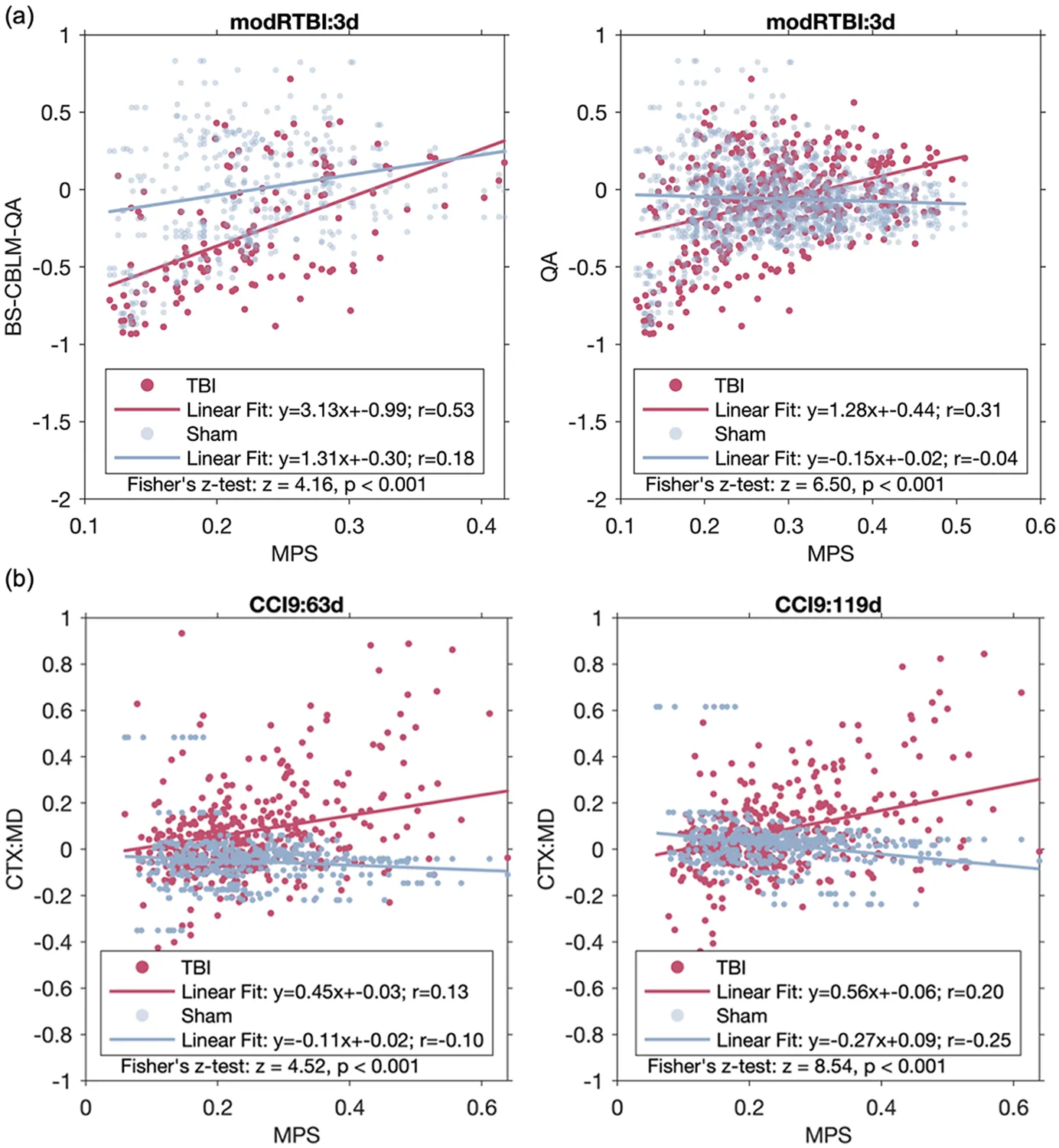
Region-wise correlation between mechanical deformation (MPS) and diffusion metric changes in rotational and CCI models. (a) Rotational TBI: Scatterplots show region-wise correlations between maximum principal strain (MPS) and diffusion metric changes at 3 days post-injury in moderate TBI (modRTBI) animals. The left panel shows QA changes in the brainstem-cerebellar region (BS-CBLM); the right panel shows whole-brain QA across all parcellated regions. (b) Controlled Cortical Impact (CCI): Region-wise correlations between MPS and mean diffusivity (MD) in cortical regions (CTX) at 63 days (left) and 119 days (right) post-injury in severe TBI (CCI9) animals. Each dot represents a parcellated brain region pooled across all subjects within the group. Statistical differences between correlation coefficients in TBI and sham groups were assessed using Fisher’s z-test, with all comparisons reaching significance (p < 0.001).

As shown above, QA changes were associated with deformation in the rotational model, while cortical MD changes in the CCI model showed a moderate association with mechanical loading at later time points. To assess the relationship between mechanical strain and post-injury microstructural changes, we pooled together the subjects in each group and performed region-wise correlation analyses between MPS and dMRI metric changes in both rotational and CCI TBI models (Fig. 11).

In the moderate rotational TBI model at 3 days post-injury, MPS showed a stronger correlation with QA changes in the BS-CBLM region in TBI animals (r = 0.53) compared to sham animals (r = 0.18), with a statistically significant difference confirmed by Fisher’s z-test (z = 4.16, p < 0.001; Fig. 11a, left). This divergence in deformation–diffusion coupling was also evident at the whole-brain level, where QA values across all parcellated regions correlated more strongly with MPS in TBI animals (r = 0.31) than in shams (r = −0.04), again reaching statistical significance (z = 6.50, p < 0.001; Fig. 11a, right).

In the CCI model, we evaluated MPS–MD relationships in cortical regions at two chronic timepoints. At 63 days post-injury, a modest positive correlation was observed in the TBI group (r = 0.13), whereas the sham group showed a weak negative correlation (r = −0.10), with the difference being significant (z = 4.52, p < 0.001; Fig. 11b, left). At 119 days, the TBI group exhibited a stronger MPS–MD correlation (r = 0.20) compared to the sham group (r = −0.25), with a highly significant group difference (z = 8.54, p < 0.001; Fig. 11b, right).

Together, these findings highlight a temporally distinct but mechanistically consistent link between mechanical strain and diffusion abnormalities across injury models. Despite differences in injury mechanism and time course, these results suggest that mechanical deformation metrics such as MPS show spatial correspondence with patterns of diffusion-inferred microstructural disruption after TBI.

## 4. Discussion

This study investigated how subject-specific biomechanical deformation predicts diffusion and structural connectivity changes in two preclinical models of TBI: rotational injury and CCI. In the rotational TBI model, moderate injury induced early diffusion changes primarily in deep brain regions, while in the CCI model, alterations emerged later but were more widespread, propagating from the cortical impact site, which is consistent with FC and structurally informed FC perturbations longitudinally (Ahmed et al., 2025). Despite differences in injury mechanism and time course, spatially specific relationships between predicted strain and MRI changes were observed in both models. These findings highlight the potential of biomechanical modeling to provide a mechanistic framework for interpreting both acute and chronic diffusion abnormalities following TBI.

In the rotational TBI model, we observed a significant increase in QA within the BS-CBLM region, which positively correlated with predicted MPS at 3 days post-injury. This result is noteworthy given that QA is often interpreted as a marker of axonal integrity—typically decreasing with fiber disruption. However, all diffusion MRI data in this study were acquired *ex vivo*, eliminating confounding factors such as edema and active inflammation. In this setting, differences between QA and DTI-derived metrics likely reflect their relative sensitivity to isotropic diffusion components, which in *ex vivo* tissue may arise from residual blood products and microstructural heterogeneity. Under these conditions, the increased QA likely reflects strain-induced microstructural changes in axonal fibers. This interpretation is consistent with the known gross pathology of sagittal-plane head rotation, which frequently produces subdural hemorrhage and extensive blood accumulation at the base of the brain and brainstem (Cullen et al., 2016). Notably, the spatial pattern of elevated QA in our data aligns with these vulnerable anatomical regions, suggesting that QA in the acute post-injury period may reflect localized mechanical effects (e.g., compression at the base of the brain) preserved in the fixed tissue. These findings emphasize the need to interpret diffusion metrics such as QA in the context of both injury mechanism and imaging conditions, particularly given that in vivo imaging may be influenced by active physiological processes (e.g., edema and inflammation) that are absent in ex vivo scans.

In contrast, the CCI model exhibited strong correlations between cortical MD and predicted deformation at 1, 63, and 119 days post-injury. The directly impacted cortex showed an increased MD and reduced FA at 1 day, followed by MD elevation and recovery of FA at later time points. This trajectory is consistent with findings from prior CCI rodent models (Zhuo et al., 2012; Mohamed et al., 2021). The delayed but persistent MD elevation aligns with the well-known secondary pathology of focal cortical impact, including inflammation, cell loss, gliosis, and breakdown of membrane barriers—all of which increase extracellular water diffusivity. As reported in earlier studies that found progressive neurodegeneration following CCI (Zhuo et al., 2012; Mohamed et al., 2021; Hall et al., 2005; Harris et al., 2016), the diffusion abnormalities originate at the site of the impact and gradually extend to include callosal and thalamic regions bilaterally. This more spatially distributed and relatively symmetric pattern likely reflects the propagation of secondary injury processes over time, rather than the focal nature of the initial mechanical insult. Importantly, the spatial distribution of cortical MD changes closely mirrored the deformation field predicted by subject-specific FE models. This correspondence suggests that long-term microstructural disruption and loss of tissue integrity were directly influenced by the initial mechanical load. In contrast, MD changes in subcortical (SCB) and brainstem-cerebellar (BS-CBLM) regions were negatively correlated with deformation—an effect also observed in sham animals—suggesting that these may reflect secondary physiological processes due to the craniectomy surgery rather than direct mechanical insult. Prior studies have demonstrated that craniectomy alone can induce significant proinflammatory responses, morphological alterations, and behavioral impairments, complicating the interpretation of injury-specific effects in experimental TBI models (Cole et al., 2011). In addition, the widespread increases in FA observed at later time points in both TBI and sham animals likely reflect developmental changes in white matter microstructure, consistent with prior longitudinal studies in pigs demonstrating age-related increases in FA during adolescent development (Ryan et al., 2018). These findings support the idea that diffusion metrics encodes both primary mechanical damage and later-stage, non-mechanical pathology, with strain prediction offering a valuable framework for disentangling the two.

Our FEM provides a novel approach to disentangling axonal injury resulting from direct mechanical loading versus axonal injury from secondary pathophysiological cascades. In the rotational TBI model, we observed weak (but significantly different from the shams) correlation between MAS and changes in SC defined by streamline counts, suggesting that axonal disruption was directly induced by mechanical forces. This finding aligns with previous neuropathological evidence in closed-head TBI models, where diffuse axonal injury (DAI)—a hallmark of human TBI across a range of severities (Johnson et al., 2016; Johnson et al., 2013; Smith and Meaney, 2000)—manifests as accumulation of axonal transport proteins such as amyloid precursor protein (APP) in swollen regions of axons (Cullen et al., 2016), In contrast, although SC metrics such as QA and streamline count effectively separated sham and TBI animals in the CCI model, these metrics did not show significant correlations with predicted deformation (MAS), suggesting they captured injury-related changes not directly driven by mechanical loading. This dissociation suggests that axonal injury in CCI likely results from secondary processes such as inflammation or delayed degeneration rather than direct deformation. However, SC matrices derived from FA and MD exhibited transient correlations with MAS—characterized by increased FA and decreased MD—at 1 day post-injury in the CCI9 group. These findings suggest that early alterations in diffusivity-based connectivity may partially reflect the immediate mechanical effects of focal impact, analogous to the strain-induced microstructural compaction observed in the BS-CBLM regions following rotational injury. These results reinforce a key mechanistic distinction between DAI in rotational TBI—which arises immediately from strain—and the slower, progressive white matter injury seen in focal impact models. Although histological studies have confirmed widespread axonal damage in CCI (Harris et al., 2016; Long et al., 2015; Mac Donald et al., 2007), our results suggest that these changes are not directly deformation-driven. This is consistent with early work by Ommaya and Gennarelli (1974), who demonstrated that rotational, but not linear, head motion generates diffuse brain injury through distributed strain fields (Ommaya and Gennarelli, 1974).

While prior studies have employed FE models to estimate regional brain deformation to compare with subsequent diffusion MRI changes, this study presents an unprecedentedly detailed comparative analysis between diffusion neuroimaging and FE model predictions. For instance, Donat et al. (2021) demonstrated in a rodent CCI model that strain and strain rate partially explained the later reduction in FA, the change in neurofilament expression, and the microglia density changes two weeks post-injury. However, DAI is a pathology difficult to replicate in lissencephalic rodents with a paucity of white matter. Similarly, Shim et al. (2022) applied subject-specific FE modeling in a sheep TBI model and linked predicted strain distributions to gross structural MRI changes but did not quantitatively assess the relationship between deformation and imaging abnormalities. In human studies, FE-simulated strain and pressure distributions have been retrospectively matched to diffusion abnormalities in TBI patients (Tan et al., 2021), and in athletes exposed to repetitive head impacts (Anderson et al., 2020; Ghajari et al., 2017; Miller et al., 2022), however, these studies often lack detailed exposure profiles. Compared to these efforts, our study provides a more comprehensive, temporally resolved analysis across two injury models, linking subject-specific FE-derived deformation fields with regional and network-level diffusion abnormalities over acute to chronic phases. By incorporating advanced diffusion metrics such as QA and modeling both streamline-based and region-based diffusion metrics, we extend previous work to reveal distinct biomechanical-imaging relationships for rotational versus focal impact. These findings highlight the value of FE models not only for injury prediction but also for interpreting the spatial and temporal complexity of post-injury dMRI abnormalities.

While this study leveraged cutting-edge dMRI, subject-specific FEM, and multi-timepoint analysis, several limitations must be acknowledged. First, although the FEM simulations incorporated individualized geometry and fiber architecture, material properties did not capture inter-subject variability. Voxel-based meshes may also introduce jagged interfaces at anatomical boundaries—such as the CSF–brain and brain–ventricle interfaces—which could theoretically lead to localized stress concentrations. To avoid potential artifacts at tissue interfaces, anatomical structures such as the sagittal sinus, falx cerebri (not prominent), and tentorium cerebelli were not modeled as two-dimensional shell elements, as done in prior studies (Giudice et al., 2021). However, recent findings indicate that mesh jaggedness at anatomical boundaries has a negligible effect on mechanical responses (Zhou et al., 2025), suggesting that this limitation likely has a minor influence on the overall conclusion. Second, while dMRI metrics like MD, FA, and QA are widely used, they are indirect markers of tissue injury and can be influenced by multiple overlapping processes such as edema, gliosis, and axonal loss. FEM offers a mechanistic framework to contextualize these changes by identifying regions of high strain. Interpreting multiple diffusion metrics together further improves biological specificity. Thus, the findings are expected to reflect robust and meaningful tissue abnormalities despite their inherent limitations. Third, the sample size, particularly within severity-stratified and longitudinal subgroups, was modest, which may have limited the statistical power of some correlation analyses. This limitation was especially pronounced in the *ex vivo* rotational injury cohort, where corresponding pre-injury scans were not available, necessitating the use of pooled sham data as a baseline reference. The lack of subject-specific reference introduces inter-subject variability that cannot be fully mitigated by increasing sample size alone, as observed in large-scale human dMRI studies (Mustafi et al., 2018; Shenton et al., 2012).

Lastly, differences in imaging conditions and cohort characteristics between the two datasets should be considered when interpreting results across models. These datasets are not intended to provide a controlled comparison but rather to illustrate how strain–diffusion relationships manifest under distinct injury and imaging conditions. The rotational dataset was acquired *ex vivo* using a 9.4T Bruker scanner, whereas the CCI dataset was acquired in vivo using a 3T GE scanner, resulting in differences in tissue state (fixed vs non-fixed), magnetic field strength, and spatial resolution. The higher spatial resolution of the ex vivo data (0.5 mm^3^) compared to the in vivo data (2 mm^3^) may introduce partial-volume effects in the latter, potentially influencing atlas registration accuracy and regional parcellation, particularly in smaller structures. In addition, the two cohorts differ in animal age and experimental design, which may further contribute to variability in injury response and diffusion measurements. These factors are known to influence the absolute magnitude of diffusion metrics. Notably, substantial differences between the in vivo and ex vivo diffusion measurements remained even in approximately age-matched sham animals (Supplementary Fig. S10), highlighting the influence of acquisition conditions and tissue state on diffusion metrics. Prior comparative studies have shown similar patterns: FA measured in *vivo* was comparable to *ex vivo* findings in formalin-fixed brains of mice (Vinh To et al., 2023), squirrel monkey (Schilling et al., 2017), and ferret (Hutchinson et al., 2017), while MD was significantly lower in *ex vivo* imaging compared to results in vivo. In a mouse TBI model (Vinh To et al., 2023), both in vivo and *ex vivo* dMRI detected injury-related effects, but in some cases, opposite trends in metrics such as FA were reported, likely reflecting the fixation effects. These divergent findings suggest that the manifestation of strain–diffusion relationships (e.g., direction and strength of correlations) may vary across imaging conditions and species. To mitigate these differences, all statistical analyses were performed independently within each dataset, with comparisons made relative to sham controls. As a result, systematic effects related to imaging conditions or biological factors would influence all animals within a dataset in a consistent manner, primarily affecting absolute metric values rather than relative injury-related changes. Cross-model comparisons were therefore restricted to qualitative patterns and relative strengths of strain–diffusion associations, rather than direct comparison of absolute diffusion values. Although the spatial correspondence between regions of mechanical strain and diffusion abnormalities observed in these animal cohorts appears robust and biologically meaningful, translating these results to human TBI requires caution due to similar limitations in imaging conditions and cohort characteristics.

Despite these limitations, this study represents the first of its kind to provide direct evidence linking subject-specific mechanical deformation fields to progressive diffusion and connectivity alterations in the brain following TBI. By aligning subject-specific deformation fields with regional and connectomic changes observed on MRI, our approach offers mechanistic insights that are difficult to extract from imaging data alone. For example, FEM predictions helped disentangle axonal injury arising from direct mechanical strain versus secondary degenerative processes—two pathologies that may produce similar diffusion signatures but differ in etiology and timing. Such interpretive clarity is essential, particularly in distinguishing DAI following rotational trauma from delayed white matter degeneration after focal impact. Beyond explanatory power, FEM-to-MRI integration has practical implications. It enables the identification of vulnerable regions based on mechanical loading patterns, guiding both injury risk assessment and therapeutic targeting. It also informs the selection and interpretation of MRI-derived biomarkers: for instance, QA and streamline-based SC metrics were shown to be more stable and deformation-sensitive than FA or MD in certain contexts. Histopathological validation would further clarify the microstructural underpinnings of these imaging-derived abnormalities and strengthen the biological interpretation of the observed strain–diffusion relationships. As imaging and modeling technologies advance, this framework can be extended to human TBI, aiding in personalized diagnostics, prognosis, and the development of protective interventions.

## Supplementary Material

1

Supplementary material associated with this article can be found, in the online version, at doi:10.1016/j.neuroimage.2026.122088.

## Figures and Tables

**Table 1:** Animal experiments and MR imaging.

	Sagittal head rotation			Controlled cortical impact				
Species	Yucatan pigs			Crossbreed Landrace piglets				
Age	6 months old			8 weeks old				
Sex	Female			Female				
Imaging Sequence	Spin-echo			Spin-echo EPI				
Scanner	Bruker 9.4T			GE 3.0T				
MRI Condition	*Ex vivo*			*In vivo*				
Samples of different severities and time of dMRI		3d	14d		3d pre	1d	63d	119d
	Sham	-	(4)	sham (6)	√	√	√	√
	mildRTBI	-	(4)	CCI3 (8)	√	√	√	√
	modRTBI	(3)	(3)	CCI9 (8)	√	√	√	√

## Data Availability

The data that support the findings of this study are available from the corresponding author upon reasonable request.
