# Supplementary material for: Mechanical deformation explains distinct neuroimaging patterns and etiologies in brain trauma
Source: Neuroimage. Author manuscript; Available in PMC 2026 Aug 19. (PMC13488385; doi:10.1016/j.neuroimage.2026.122088)
Supplement: Supplementary file 1 [file NIHMS2200360-supplement-1.docx]

Supplementary Materials for

**Mechanical Deformation Explains Distinct Neuroimaging Patterns and Etiologies in Brain Trauma**

Yuan Huang *et al.*

*Corresponding author: Taotao Wu, taotao.wu@uga.edu

**This PDF file includes:**

Tables S1 to S4

Figs. S1 to S10

Table S1. Body weight, brain weight, and gross pathology findings at brain extraction in the rotational injury cohort.

| **Pig ID** | **Type of injury** | **Body weight**  **(kg)** | **Survival Time (d)** | **Brain weight (g)** | **Gross Pathology Findings** |
| --- | --- | --- | --- | --- | --- |
| W1 | Sham | 25 | 14 | 82.0 | No gross abnormalities observed |
| W2 | mildRTBI | 25 | 14 | 81.0 | Brain grossly unremarkable |
| W3 | mildRTBI | 29 | 14 | 78.7 | Brain grossly unremarkable |
| W4 | modRTBI | 30 | 14 | 85.4 | Small focal subdural hemorrhage over posterior lobe and at skull base near pituitary |
| W5 | Sham | 25 | 14 | 82.8 | No gross abnormalities observed |
| W6 | Sham | 27 | 14 | 83.1 | No gross abnormalities observed |
| W7 | Sham | 28 | 14 | 78.3 | No gross abnormalities observed |
| W8 | mildRTBI | 27 | 14 | 94.1 | Brain grossly unremarkable |
| W10 | modRTBI | 25 | 14 | 77.0 | Mild subdural discoloration consistent with resolving hemorrhage (possible hemosiderin deposition) |
| W11 | mildRTBI | 25 | 14 | 83.7 | No clear focal hemorrhage observed |
| W12 | modRTBI | 27 | 14 | 88.0 | Very mild hemosiderin staining |
| W13 | modRTBI | 32 | 3 | 86.6 | Major acute subdural hemorrhage at extraction (Fig. S1) |
| W14 | modRTBI | 34 | 3 | 83.8 | Moderate subdural hemorrhage (Fig. S1) |
| W15 | modRTBI | 36 | 3 | 88.8 | Tentorial hemorrhage; no additional abnormalities |

Table S2. Body weight and brain size across timepoints in the CCI cohort.

| **Pig ID** | **Type of injury** | **Body weight**  **(kg)** | | | | **Intracranial volume***  **(mL)** | | | | **Ex vivo brain weight (g)** |
| --- | --- | --- | --- | --- | --- | --- | --- | --- | --- | --- |
|  |  | **3d pre** | **1d** | **63d** | **119d** | **3d pre** | **1d** | **63d** | **119d** |  |
| P2 | CCI9 | 18 | 18 | 66 | 91 | 112 | 112 | 131 | 122 | 105 |
| P3 | CCI3 | 15 | 15 | 69 | 96 | 115 | 103 | 131 | 153 | 106 |
| P4 | CCI9 | 22 | 22 | 69 | 88 | 119 | 116 | 135 | 151 | 105 |
| P5 | Sham | 23 | 23 | 78 | 100 | 125 | 114 | 148 | 151 | 114 |
| P6 | CCI3 | 17 | 17 | 70 | 91 | 120 | 108 | 151 | 141 | 111 |
| P13 | Sham | 21 | 21 | 74 | 101 | 99 | 107 | 156 | 163 | 113 |
| P14 | CCI3 | 22 | 22 | 72 | 95 | 105 | 77 | 143 | 149 | 103 |
| P15 | CCI3 | 27 | 27 | 81 | 94 | 103 | 108 | 142 | 150 | 104 |
| P16 | Sham | 29 | 29 | 78 | 93 | 107 | 100 | 150 | 156 | 113 |
| P17 | CCI9 | 27 | 27 | 79 | 102 | 107 | 107 | 138 | 155 | 100 |
| P18 | CCI9 | 25 | 25 | 77 | 100 | 105 | 109 | 144 | 163 | 105 |
| P19 | Sham | 18 | 18 | 58 | 72 | 99 | 103 | 151 | 148 | 107 |
| P20 | CCI3 | 26 | 26 | 70 | 90 | 91 | 98 | 136 | 148 | 102 |
| P21 | CCI3 | 21 | 21 | 69 | 89 | 103 | 99 | 145 | 149 | 109 |
| P22 | CCI9 | 30 | 30 | 82 | 108 | 102 | 108 | 138 | 148 | 99 |
| P23 | CCI9 | 20 | 20 | 64 | 82 | 104 | 101 | 129 | 144 | 96 |
| P24 | Sham | 20 | 20 | 62 | 75 | 101 | 97 | 140 | 154 | 104 |
| P25 | CCI3 | 23 | 23 | 73 | 95 | 104 | 97 | 141 | 152 | 104 |
| P26 | CCI3 | 21 | 21 | 65 | 88 | 99 | 101 | 137 | 153 | 101 |
| P27 | Sham | 25 | 25 | 75 | 99 | 105 | 97 | 141 | 155 | 112 |
| P28 | CCI9 | 23 | 23 | 70 | 89 | 99 | 102 | 135 | 150 | 99 |
| P29 | CCI9 | 22 | 22 | 67 | 87 | 110 | 109 | 143 | 161 | 108 |

*Intracranial volume (ICV) was calculated from the T1-weighted brain mask as voxel count$\times$voxel volume.

Table S3. Region grouping of the Saikali pig brain atlas

| **Group** | **Region ID (s)** |
| --- | --- |
| **CTX**  **(n=54)** | 99: Primary Somatosensory Cortex; 100: Primary Motor Cortex; 101: Somatosensory Association Cortex; 102: Premotor Cortex; 103: Dorsolateral prefrontal cortex; 104: Anterior prefrontal cortex;105: Orbitofrontal cortex; 106: Insular cortex; 107: Primary visual cortex (V1); 108: Secondary visual cortex (V2); 109: Associative visual cortex (V3); 110: Inferior temporal gyrus; 111: Middle temporal gyrus; 112: Superior temporal gyrus; 113: Ventral Posterior cingulate cortex; 114: Ventral Anterior cingulate cortex; 115: Ectosplenial area; 116: Pyriform cortex; 117: Retrosplenial cingular cortex; 118: Dorsal posterior cingular cortex; 119: Dorsal anterior cingulate cortex; 120: Anterior entorhinal cortex; 121: Perirhinal cortex; 122: Parahippocampal cortex; 123: Fusiform gyrus; 124: Auditory Cortex; 125: Prepyriform area; 151: Primary Somatosensory Cortex; 152: Primary Motor Cortex; 153: Somatosensory Association Cortex; 154: Premotor Cortex; 155: Dorsolateral prefrontal cortex; 156: Anterior prefrontal cortex; 157: Orbitofrontal cortex; 158: Insular cortex; 159: Primary visual cortex (V1); 160: Secondary visual cortex (V2); 161: Associative visual cortex (V3); 162: Inferior temporal gyrus; 163: Middle temporal gyrus; 164: Superior temporal gyrus; 165: Ventral Posterior cingulate cortex; 166: Ventral Anterior cingulate cortex; 167: Ectosplenial area; 168: Pyriform cortex; 169: Retrosplenial cingular cortex; 170: Dorsal posterior cingular cortex; 171: Dorsal anterior cingulate cortex; 172: Anterior entorhinal cortex; 173: Perirhinal cortex; 174: Parahippocampal cortex; 175: Fusiform gyrus; 176: Auditory Cortex; 177: Prepyriform area |
| **SCB**  **(n=57)** | 1: Posterior commissure (pc); 2: Anterior commissure (ac); 3: Optic chiasm (ox); 4: Corpus callosum (cc); 5: Anteroventral thalamic nucleus (AV); 6: Anteroventral thalamic nucleus (AV); 7: Central thalamic area (CTA); 8: Central thalamic area (CTA); 9: Mediodorsal thalamic nucleus (MD); 10: Mediodorsal thalamic nucleus (MD); 11: Laterodorsal thalamic nucleus (LD); 12: Laterodorsal thalamic nucleus (LD); 13: Geniculate nuclei (G); 14: Geniculate nuclei (G); 15: Pulvinar nuclei (Pul); 16: Pulvinar nuclei (Pul); 17: Reticular thalamic nucleus (Rt); 18: Reticular thalamic nucleus (Rt); 19: Ventral anterior thalamic nucleus (VA); 20: Ventral anterior thalamic nucleus (VA); 21: Ventral posterior thalamic nucleus (VP); 22: Ventral posterior thalamic nucleus (VP); 23: Accumbens nucleus (Acb); 24: Accumbens nucleus (Acb); 25: Caudate nucleus (Cd); 26: Caudate nucleus (Cd); 27: Claustrum (Cl); 28: Claustrum (Cl); 29: Globus pallidus (GP); 30: Globus pallidus (GP); 31: Putamen (Pu); 32: Putamen (Pu); 33: Substantia nigra (SN); 34: Substantia nigra (SN); 35: Fornix (fx); 36: Hippocampus (Hi); 37: Hippocampus (Hi); 38: Subiculum (S); 39: Subiculum (S); 40: Amygdala (A); 41: Amygdala (A); 42: Subthalamic nucleus (STh); 43: Subthalamic nucleus (STh); 44: Zona incerta (ZI); 45: Zona incerta (ZI); 46: Habenular nuclei (Hb); 47: Habenular nuclei (Hb); 48: Pineal gland (Pi); 49: Anterior hypothalamic area (AH); 50: Anterior hypothalamic area (AH); 51: Mamillary body (MB); 52: Medial hypothalamic area (MH); 53: Medial hypothalamic area (MH); 94: Lateral ventricle (LV); 95: Lateral ventricle (LV); 96: Third ventricle (3V); 98: Cerebral aqueduct (Sylvius) (Aq) |
| **BS-CBLM**  **(n=65)** | 54: Inferior colliculus (IC); 55: Inferior colliculus (IC); 56: Superior colliculus (SC); 57: Superior colliculus (SC); 58: Periaqueductal gray (PAG); 59: Red Nucleus (R); 60: Red Nucleus (R); 61: Nucleus of the optic tract (OT); 62: Nucleus of the optic tract (OT); 63: Pretectal area (PTc); 64: Pretectal area (PTc); 65: Interpeduncular nucleus (IP); 66: Pontine nuclei (Pn); 67: Pontine nuclei (Pn); 68: Reticulotegmental nucleus of the pons (RFTg); 69: Reticulotegmental nucleus of the pons (RFTg); 70: Cuneate nucleus (Cu); 71: Cuneate nucleus (Cu); 72: Nucleus of the solitary tract (Sol); 73: Nucleus of the solitary tract (Sol); 74: Olivary nuclei (Ol); 75: Olivary nuclei (Ol); 76: Superior olivary complex (SOC); 77: Superior olivary complex (SOC); 78: Dorsal motor nucleus of vagus (10); 79: Dorsal motor nucleus of vagus (10); 80: Motor trigeminal nucleus (Mo5); 81: Motor trigeminal nucleus (Mo5); 82: Spinal trigeminal nucleus (Sp5); 83: Spinal trigeminal nucleus (Sp5); 84: Abducens nucleus (6); 85: Abducens nucleus (6); 86: Facial nucleus (7); 87: Facial nucleus (7); 88: Hypoglossal nucleus (12); 89: Hypoglossal nucleus (12); 90: Spinal trigeminal tract (sp5); 91: Spinal trigeminal tract (sp5); 92: Vestibular nucleus (Ve); 93: Vestibular nucleus (Ve); 97: Fourth ventricle (4V); 127: Lateral cerebellar nucleus (Lat); 128: Lateral cerebellar nucleus (Lat); 129: Medial cerebellar nucleus (Med); 130: Medial cerebellar nucleus (Med); 131: Interposed cerebellar nucleus (Int); 132: Interposed cerebellar nucleus (Int); 133: Inferior cerebellar peduncle (ICP); 134: Inferior cerebellar peduncle (ICP); 135: Medial cerebellar peduncle (MCP); 136: Medial cerebellar peduncle (MCP); 137: Superior cerebellar peduncle (SCP); 138: Superior cerebellar peduncle (SCP); 139: Cerebellar lobule I,II; 140: Cerebellar lobule III; 141: Cerebellar lobule IV; 142: Cerebellar lobule V; 143: Cerebellar lobule VI; 144: Cerebellar lobule VIIB; 145: Cerebellar lobule VIIIA; 146: Cerebellar lobule IX; 147: Cerebellar lobule X; 148: Crus 1 of the ansiform lobule; 149: Crus 2 of the ansiform lobule; 150: Cerebellar lobule VIIIB |
| **Excluded**  **(N=2)** | 126&178: Olfactory bulb |

Table S4. Material properties used in the finite element model

| **Part** | **Elements** | **Element type** | **Element Formulation** | **Hourglass control** | **Constitutive Model** | **Material Parameters** |
| --- | --- | --- | --- | --- | --- | --- |
| **CSF and Ventricles** | 18,701 | Voxel (1 mm^3^) | Constant stress element | N/A | Linear Viscoelastic | $\rho=1.04\times{10}^{-6} kg/mm^{3}$  $K=2.19 GPa$  $G_{0}=5.0\times{10}^{-7} GPa$  $G_{\infty}=1.0\times{10}^{-7} GPa$  $\tau=0.0125 ms$ |
| **Brain Parenchyma** | 81,593 |  |  | IHQ = 3^*^ | Hyper-Viscoelastic | $\rho=1.23\times{10}^{-6} kg/mm^{3}$  $\mu^{\dagger}=1.0\times{10}^{-6} GPa$  $\alpha^{\dagger}=5.1$  $G_{1}=0.568; \beta=10 ms^{-1}$  $G_{2}=0.123; \beta=1 ms^{-1}$  $G_{3}=0.084; \beta=0.1 ms^{-1}$  $G_{4}=0.045; \beta=0.01 ms^{-1}$  $G_{4}=0.04; \beta=0.0005 ms^{-1}$  $\nu=0.495$ |
| **Axonal Tracts** | 636,930^‡^ | Beam (~ 1 mm) | Hughes-Liu with cross section integration | N/A | Linear Elastic | $E=3.0\times{10}^{-6} GPa$  $\rho=1\times{10}^{-10} kg/mm^{3}$  $\nu=0.45$ |
| **Dura** | 18,844 | Quadrilateral  (1 mm^2^) | Belytshko-Tsay Elements | N/A | Rigid | $\rho=2.09\times{10}^{-6} kg/mm^{3}$ |

^*^IHQ = 3; Flanagan-Belytshko viscous form with exact volume integration for solid elements

^†^Ogden parameters ($\mu$ and $\alpha$) represent the quasi-static response

^‡^Element counts for axonal tracts are subject-specific; values shown represent a single subject and are provided to illustrate relative scale.

**Figure S1. Representative gross pathology in moderate rotational TBI animals at 3 days post-injury.** Extra-axial hemorrhage was observed predominantly along the dorsal midline and tentorial regions. No intraparenchymal bleeding was identified.

**Figure S2: Representative overlays between MRI-derived brain mask surfaces (red) and morphed FE model surfaces (green) for representative subjects from the CCI (P2) and rotational TBI (W2) cohorts**. Orthogonal views are shown to illustrate the anatomical alignment achieved by the non-linear morphing framework.

**Figure S3.** **Effect of embedded fiber architecture on predicted brain strain distributions.** Representative slices of maximum principal strain (MPS) are shown for models without fibers (left column), with embedded fibers (middle column), and their differences (right column) for (top row) a controlled cortical impact (CCI) case and (bottom row) a rotational TBI (modRTBI) case. Warm colors indicate higher strain magnitudes. Difference maps (no-fiber minus fiber) highlight localized changes introduced by fiber inclusion. While overall strain patterns remain similar, fiber architecture introduces spatially heterogeneous but low-amplitude variations, with differences on the order of ~0.01 relative to strains of ~0.1. Separate color scales are used for CCI, rotational, and difference maps.

**Figure S4.** **Parcellation atlas mapped onto the finite element (FE) brain model**. The atlas was registered to the FE template space using co-registered T1- and T2-weighted structural images.

**Fig. S5. Individual structural connectivity (SC) matrices for the rotational cohort.**
Each matrix represents pairwise connectivity (FA, QA, MD, RD, AD, and streamline-count connectivity) between the 54 cortical ROIs. These matrices are provided to illustrate inter-subject variability and support the edgewise analyses reported in the main text. **Panels continue on the following pages.**

**Fig. S5.** **(continued).**

**Fig. S5.** **(continued).**

**Fig. S5.** **(continued).**

**Fig. S5.** **(continued).**

**Fig. S5.** **(continued).**

**Fig. S6. Edgewise comparisons of structural connectivity (SC) matrices in the rotational TBI model.** Each matrix shows edgewise group differences between TBI and sham animals for a given diffusion metric (FA, QA, MD, RD, AD) or streamline count at specific time points (3 or 14 days post-injury) for mild (mTBI) and moderate (moTBI) cases. The lower triangle of each matrix displays the mean difference in connectivity strength (TBI – sham), while the upper triangle shows uncorrected p-values from two-sample t-tests between the TBI and sham groups. Only a small number of edges showed significant group differences, with QA and streamline count appearing more stable across conditions.

**Fig. S7. Individual structural connectivity (SC) matrices for the CCI cohort.**
Each matrix represents pairwise connectivity (FA, QA, MD, RD, AD, and streamline-count connectivity) between the 54 cortical ROIs. Rows correspond to individual subjects, and columns correspond to time points. These matrices are provided to illustrate inter-subject variability and support the edgewise analyses reported in the main text. **Panels continue on the following pages.**

**Fig. S7.** **(continued).**

**Fig. S7.** **(continued).**

**Fig. S7.** **(continued).**

**Fig. S7.** **(continued).**

**Fig. S7.** **(continued).**

**Fig. S7.** **(continued).**

**Fig. S7.** **(continued).**

**Fig. S7.** **(continued).**

**Fig. S7.** **(continued).**

 **Fig. S7.** **(continued).**

 **Fig. S7.** **(continued).**

 **Fig. S7.** **(continued).**

**Fig. S7.** **(continued).**

**Fig. S7.** **(continued).**

**Fig. S7.** **(continued).**

**Fig. S7.** **(continued).**

**Fig. S7.** **(continued).**

**Fig. S7.** **(continued).**

**Fig. S7.** **(continued).**

**Fig. S7.** **(continued).**

**Fig. S7.** **(continued).**

**Fig. S7.** **(continued).**

**Fig. S7.** **(continued).**

**Fig. S7.** **(continued).**

**Fig. S8. Edgewise comparisons of structural connectivity (SC) matrices in the CCI TBI model.** Each matrix shows edgewise group differences between TBI and sham animals for a given diffusion metric (FA, QA, MD, RD, AD) or streamline count at specific time points (1, 63 or 119 days post-injury) for moderate (moTBI) and severe (sTBI) cases. The lower triangle of each matrix displays the mean difference in connectivity strength (Post-injury – Pre-injury), while the upper triangle shows FDR-corrected p-values from two-sample t-tests between the post-injury and pre-injury groups. Only a small number of edges showed significant group differences, with QA and streamline count appearing more stable across conditions. **Panels continue on the following page**.

Fig. S8. (continued).

mildRTBI

**Fig. S9. Spatial variability of diffusion and strain metrics.** Voxel-wise mean and standard deviation maps are shown for diffusion metrics (ΔMD, ΔQA, ΔFA) and strain measures (MPS, MAS). Δ maps represent absolute changes relative to baseline (post–pre for CCI; subject–sham mean for rotational injury). **Panels continue on the following pages.**

modRTBI (3d)

modRTBI (14d)

Fig. S9. (continued).

CCI3

CCI9

Sham

Fig. S9. (continued).

Figure S10. *Ex vivo* and *in vivo* diffusion metric comparison in age-matched sham pigs. Group mean diffusion maps and region-wise ROI comparisons are shown for FA, MD, and QA. *Ex vivo* data were from 6-month-old Yucatan sham pigs scanned after sacrifice, and *in vivo* data were from Landrace sham pigs at the final imaging time point (5.7 months). Because the absolute metric ranges differed substantially between *ex vivo* and *in vivo* acquisitions, map intensities are displayed with separate color axes. In the scatter plots, each node represents the group-mean value for a specific region. Region-wise correlations between *ex vivo* and *in vivo* measurements were weak for FA and MD and modest for QA, indicating that systematic acquisition/specimen differences remain despite reduced age-related confounding.
